# Sirt3 improves monosodium urate crystal-induced inflammation by suppressing Acod1 expression

**DOI:** 10.1186/s13075-023-03107-6

**Published:** 2023-07-19

**Authors:** Linxi Lv, Hui Jiang, Dianze Song, Xiaoqin Zhou, Feng Chen, Long Ren, Yongen Xie, Mei Zeng

**Affiliations:** 1 Institute of Rheumatology and Immunology, the Affiliated Hospital of North Sichuan Medical College, 1# South Maoyuan Road, Nanchong, 637001 Sichuan China; 2 Medical Imaging Key Laboratory of Sichuan Province, the Affiliated Hospital of North Sichuan Medical College, 1# South Maoyuan Road, Nanchong, 637001 Sichuan China; 3 Institute of Basic Medicine and Forensic Medicine, North Sichuan Medical College, 234# Fujiang Road, Nanchong, 637000 Sichuan Province China; 4 The Fifth People’s Hospital of Nanchong City, 21# Bajiao Street, Nanchong, 637100 Sichuan China

**Keywords:** Sirt3, MSU crystals, SOD2 acetylation level, Acod1, TCA cycle

## Abstract

**Background:**

Previous studies have revealed that Sirt3 deficiency is associated with several inflammatory responses. The purpose of this study is to investigate the role and potential molecular mechanisms of Sirt3 in the inflammation induced by monosodium urate (MSU) crystals.

**Methods:**

The Sirt3 expression level in the peripheral blood mononuclear cells (PBMCs) of patients with gout was measured. Function and molecular mechanism of Sirt3 in MSU crystal-induced inflammation were investigated in bone marrow-derived macrophages (BMDMs), C57BL/6 mouse, and Sirt3^−/−^ mouse.

**Results:**

Sirt3 expression was decreased in the PBMCs of patients with gout. Sirt3 agonist (Viniferin) inhibited the acetylation levels of mitochondrial proteins including the SOD2 protein. RNA sequencing, bio-informatics analysis, RT-PCR, and Western blot demonstrated that Sirt3 could suppress the expression of Acod1 (Irg1), which plays an important role in gout. In BMDMs treated with palmitic acid (C16:0) plus MSU crystals, Acod1 knockdown repressed mitochondrial reactive oxygen species (mtROS) over-production, macrophage migration, and mitochondrial fragmentation, and Acod1 improved AMPK activity. The over-expression of Acod1 did not significantly affect the level of itaconic acid, but greatly decreased the levels of some important intermediate metabolites of the tricarboxylic acid (TCA) cycle. These data indicate that Acod1 exerts a pro-inflammatory role in MSU crystal-induced inflammation and is independent of the metabolic level of itaconic acid. Sirt3 deficiency exacerbates inflammatory response induced by MSU crystals in vitro and in vivo.

**Conclusion:**

The current study has shown that Sirt3 can alleviate the MSU crystal-induced inflammation by inhibiting the expression of Acod1.

**Supplementary Information:**

The online version contains supplementary material available at 10.1186/s13075-023-03107-6.

## Background

Gout is a common form of inflammatory arthritis caused by the deposition of MSU crystals. Elevated levels of uncontrolled serum urate levels usually result in the deposition of MSU crystals, which are the main reason for acute gout flares, and recurrent attacks of gout are inevitably destructive to the joints. MSU crystals stimulate the innate immune system and trigger strong inflammatory responses to the joints and periarticular tissues [1, 2]. However, MSU crystals are present in some patients with asymptomatic hyperuricemia [3] or past gout attacks, but may not induce acute inflammatory responses [4]. This indicates that the MSU crystal-induced clinical manifestation is a process dependent on many factors.

It has been well known that MSU crystals trigger inflammatory arthritis by activating the NLRP3 inflammasome [5]. The MSU crystals, as the second signal, interact with the resident macrophages to activate the NLRP3 inflammasome and lead to the release of bio-active IL-1β [5]. Saturated long-chain fatty acids, such as palmitic acid (C16:0) and stearic acid (C18:0), can provide this first signal and cooperate with MSU crystals to induce large amounts of mature IL-1β [6, 7]. Inhibition of mitochondrial ROS driven by fatty acids in macrophages can reduce acute gout inflammation [8]. MSU crystals also stimulated the production of ROS [9]. Furthermore, ROS accumulation plays an important role in mediating gout inflammation [10]. There is increasing evidence that ROS is involved in NF-κB signal transduction [11]. Excessive ROS can cause mitochondrial dysfunction which is closely associated with NLRP3 inflammasome activation.

SIRT3 is mainly located in mitochondria [12, 13], and its deficiency is related to mitochondrial dysfunction and redox steady state [14, 15]. It has been reported that SIRT3 regulates the acetylated state of various metabolic enzymes and proteins involved in oxidative phosphorylation in mitochondria [16–18]. Sirt3 reduces mtROS by activating SOD2 (superoxide dismutase 2, mitochondria) [19]. Increased expression and activity of SIRT3 reduce mitochondrial dysfunction and fragmentation [20]. The expression/activation level of SIRT3 in macrophages can control the activation of NLRP3 inflammasome [21]. It is reported that loss of SIRT3 activity exists in various pathology in aging [22] and multiple pathologies, including cardiovascular diseases, diabetes, and pulmonary arterial hypertension [17–19]. Depletion of Sirt3 leads to the inhibition of β oxidation of long-chain fatty acids, such as palmitic acid [17]. The emphasis of this study is whether SIRT3 is involved in inflammation induced by Palmitic (C16:0) plus MSU crystals and its molecular mechanism.

## Methods and materials

### Patients

All participants were gout patients from the Department of Rheumatology of The Affiliated Hospital of North Sichuan Medical College. Patients with gouty arthritis (GA) must conform to the classification criteria in the 1977 American College of Rheumatology classification criteria. GA patients were further divided into the acute gout (AG) group (swelling, redness, stiffness, and severe pain within the past 2 weeks) and intercritical gout (IG) group (no swelling, redness, stiffness, and severe pain within the past 2 weeks) according to whether the patients showed symptoms of onset at the joint or not. Peripheral blood mononuclear cells (PBMCs) from patients were isolated with Ficoll-Paque PLUS (GE Healthcare, Piscataway, NJ, USA) following the manufacturer’s instructions. The cells were used for RNA and protein extraction. This study was approved by the Ethics Committee of the Affiliated Hospital of North Sichuan Medical College, and all patients must provide informed consent to participate in the study. Information of gout patients and health controls is shown in Table 1.

**Table 1:** The characteristics of gout patients and healthy controls are listed in this study

**Items**	AG (*n* = 30)	IG (*n* = 30)	*P* value
**UA** (μmol/L)	447.7 ± 75.02	546.2 ± 124.1	< 0.05
**ESR** (mm/h)	30.10 ± 20.73	9.99 ± 7.829	< 0.05
**CRP** (mg/L)	22.94 ± 22.91	2.31 ± 1.99	< 0.05
**WBC** (10^9^/L)	9.34 ± 3.06	6.92 ± 1.95	< 0.05
**GR** (10^9^/L)	6.45 ± 2.88	4.35 ± 1.71	< 0.05
**MO** (10^9^/L)	0.58 ± 0.25	0.40 ± 0.11	< 0.05
**PLT** (10^9^/L)	230.1 ± 74.02	189.0 ± 50.78	< 0.05
LY (10^9^/L)	2.13 ± 0.69	2.00 ± 0.61	0.24
Age (year)	41.11 ± 13.46	39.30 ± 13.28	0.65
BMI (Kg/m^2^)	24.47 ± 2.70	24.53 ± 1.72	0.86

*AG* Acute gout, *IG* Intercritical gout, *UA* Blood uric acid concentration, *ESR* Erythrocyte sedimentation rate, *CRP* C-reaction protein, *WBC* White blood cell count, *GR* Neutrophil granulocyte absolute value, *MO* Monocyte count, *PLT* Blood platelet count, *LY* Lymphocyte count, *BMI* Body mass index

### Palmitic acid and albumin conjugation

Palmitic acid (C16:0) was prepared according to the method described by Christopher et al. [8]. Palmitic acid (C16:0; Sigma-Aldrich; PO500) was dissolved in 100 mM NaOH solution at 90 °C to 50 mM, and then diluted 1: 5 with 5% BSA solution at 37 °C. BSA-conjugated C16:0 was filtered with a 0.2-μm acrodisc syringe filter and stored at − 20 °C.

### Reagents

HY-133987, Mito-TEMPO, and Viniferin were purchased from MedChemExpress (MCE). The formation of the MSU crystals is performed in accordance with the following method as described previously [5]. In 400 mL of sterile water, 1.0 g of uric acid (Sigma) and 0.48 g of sodium hydroxide were dissolved. The pH was adjusted to 8.9 and the solution is fully dissolved by heating and stirring at 120 °C. The mixture was crystallized at 4 °C overnight. LPS contamination was detected by the Limulus amoebocyte lysate assay.

### Isolation and culture of BMDMs, siRNA, or plasmids transfection

Bone marrow-derived macrophages (BMDMs) were isolated from C57BL/6 or Sirt3^−/−^ mice (regardless of sex) at 8 to 10 weeks of age. Macrophages were cultured in high glucose DMEM supplemented with 10% fetal bovine serum (FBS), M-CSF (10 ng/mL), and 100 U/mL penicillin–streptomycin for 5–7 days. SiRNA targeting Acod1 were synthesized by Sangon Biotechnology and their sequences are shown in Table 2. The open reading frame (ORF) of mouse Acod1 (NM_008392) was cloned into a pcDNA3.1 vector and sequencing was performed to eliminate mutations. SiRNA and plasmid transfection were performed using LipoRNAi and Lipo8000 kits (Beyotime, China), respectively, according to the manufacturer’s instructions.

**Table 2:** The targeted siRNA sequence for mice Acod1

Gene	**Sense (5′-3′)**	**Antisense (5′-3′)**
Negative control	UUCUCCGAACGUGUCACGUTT	ACGUGACACGUUCGGAGAATT
Mice Acod1	CCUGACGUCCAGUACGUAATT	UUACGUACUGGACGUCAGGTT

### Enzyme-linked immunosorbent assay (ELISA)

The culture supernatants of BMDMs and mouse peritoneal fluid were used to test IL-1β secretion by ELISA according to the instructions (NEOBIOSCIENCE, China).

### RNA-sequencing and bio-informatics analysis

The total RNA is isolated in BMDMs of Sirt3^+/+^ and Sirt3^−/−^ treated with C16:0 + MSU in triplicate. Transcriptome sequencing and subsequent bio-informatics analysis were carried out by the Applied Protein Technology Co., Ltd. (Shanghai, China). The mRNA was purified from 1 μg total RNA using oligo (dT) magnetic beads followed by fragmentation in the ABclonal First Strand Synthesis Reaction Buffer. Subsequently, using mRNA fragments as templates, the first strand of cDNA is synthesized using random primers and Reverse Transcriptase (RNase H), followed by the second strand of cDNA synthesis using DNA polymerase I, RNAseH, buffer, and dNTPs. The synthesized double-stranded cDNA fragments are ligated to the linker sequence for PCR amplification. The PCR product was purified and library quality was evaluated using Agilent Bioanalyzer 4150 system. Finally, the library preparations were sequenced on an Illumina Novaseq 6000 (or MGISEQ-T7), and 150 bp paired-end reads were generated. The data generated from Illumina (or BGI) platform were used for bioinformatics analysis. Differential expression genes (DEGs) analysis was performed using the DESeq2, DEGs with | log2FC |> 1 represent upregulation, DEGs with | log2FC |< − 1 mean downregulation, and Padj < 0.05 were considered to be significantly differentially expressed genes.

### Quantitative PCR (qPCR)

Total RNA was extracted from macrophages using the TRIzol reagent (Invitrogen) and reverse-transcribed into the cDNA using the reverse transcription reagent (TaKaRa, China). QPCR was performed using SYBR Green PCR Master Mix (Vazyme, China) and *GAPDH* as an internal reference. Target gene expression was analyzed through the 2^−△△CT^ method. Primer sequences are shown in Table 3.

**Table 3:** The primers used for quantitative PCR

Items	**Forward sequence (5′-3′)**	**Reverse sequence (5′-3′)**
Mice IL-1β	GAAATGCCACCTTTTGACAGTG	TGGATGCTCTCATCAGGACAG
Mice Nlrc3	CCACGAGGAAGCAAGAGGTG	TTTGTCCCGGACTTGTAGCAG
Mice Ccl2	CCTTTGAATGTGAAGTTGACCCG	TAAGGCATCACAGTCCGAGTCAC
Mice iNOS	GGAGTGACGGCAAACATGACT	TCGATGCACAACTGGGTGAAC
Mice Itgb7	CTCAATGAAGGACGACTTGGAAC	GACAACGCTCCAGTCGGCTG
Mice Acod1	CCCATAGCGAACGCTGCCACT	GAAGGCACCGAACCCTGACCC
Mice COX-2	GGAGTGACGGCAAACATGACT	TCAGGAAGCTCCTTATTTCCCTT
Mice TNF-α	ACTGAACTTCGGGGTGATCG	TCCACTTGGTGGTTTGTGAGT
Mice Sirt3	GAGCGGCCTCTACAGCAAC	GGAAGTAGTGAGTGACATTGGG
Mice GAPDH	AGGTCGGTGTGAACGGATTTG	GGGGTCGTTGATGGCAACA
Human Sirt3	ACCCAGTGGCATTCCAGAC	GGCTTGGGGTTGTGAAAGAAG
GAPDH	CTGGGCTACACTGAGCACC	AAGTGGTCGTTGAGGGCAATG

### Western blot

BMDMs were treated with different intervention methods and then lysed in RIPA buffer. The collected mouse paws were homogenized in RIPA buffer solution. After cells and tissues were lysed and centrifuged, the supernatants were used for Western blot analysis. Nuclear protein extraction was performed using the Nuclear and Cytoplasmic Protein Extraction Kit (Beyotime, China). Protein levels in different groups are represented as ratios to corresponding internal reference protein levels. The band intensity was quantified using the Image J software. All Western blot assays show one representative out of three in this study.

### Detection of mitochondrial ROS generation

Mitochondrial ROS levels were detected using Mito-SOX (Invitrogen, M36008). BMDMs were stained with a Mito-SOX probe according to instructions and then were detected using the laser confocal microscope (LCM) or flow cytometer (FCM). By LCM detection, the final concentration of Mito-SOX is 2.5 μM. The final concentration of Mito-SOX was 1.25 μM through FCM assay.

### Determination of mitochondrial SOD activity

According to the manufacturer’s instructions, the SOD activity of BMDMs was detected using Cu/ZnSOD and Mn-SOD Assay Kit of WST-8 (Beyotime Biotechnology, Shanghai, China). Briefly, Mitochondrial proteins were extracted and the protein concentration was measured by the BCA assay kit.

### Antibodies

All antibodies used for Western blot and immunofluorescence were described in Table 4.

**Table 4:** The antibodies were used in Western blot and immunofluorescence

Antibody	Source	Identifier	Dilution
Sirt3	HUABIO	R1511-3	1:750
ACOD1	Abcam	ab222411	1:1000
Acetylated-lysine antibody	Cell Signaling Technology (CST)	9441	1:1500
Anti-SOD2/MnSOD (acetyl K68)	Abcam	ab137037	1:1000
Integrin beta 7	Proteintech	11,328–1-AP	1:1000
NF-κB p65	HUABIO	ET1603-12	1:1000
Phospho-NF-κB p65 (Ser536) (93H1)	Cell Signaling Technology (CST)	3033S	1:750
Phospho-DRP1 (Ser616)	Cell Signaling Technology (CST)	3455S	1:750
AMPK alpha 1	HUABIO	ET1608-40	1:1000
Phospho-AMPKα (Thr172) (D4D6D)	Cell Signaling Technology (CST)	2535S	1:750
CCL2	HUABIO	HA500267	1:1000
CCR2	HUABIO	ET1611-65	1:1000
iNOS	Abcam	ab178945	1:1500
COX2/Cyclooxygenase 2	HUABIO	ET1610-23	1:1500
NLRP3(NLRC3)	HUABIO	ET1610-93	1:1500
IL-1β (D3U3E)	Cell Signaling Technology (CST)	12703S	1:1000
Caspase-1	Adipogene	AG-20B-0042-C100	1:1000
Myeloperoxidase (MPO)	Abcam	ab208670	1:100
ASC(WB)	Adipogene	AG-25B-0006PF-C100	1:1000
ASC(IF)	Santa Cruz Biotech	Sc-514414	1:50
Tubulin	HUABIO	ET1602-4	1:1500
GAPDH	HUABIO	HA721136	1:1500

### Quantitative analysis of mitochondrial respiration

Cellular respiration was detected using Seahorse XF24 extracellular flux analyzer (Agilent Seahorse Biosciences). Each well was seeded 20,000 cells. The cells were cultured in DMEM medium and replaced with a preheated XF assay medium for 1 h. O_2_ consumption rate (OCR) was tested in the Seahorse XF24 analyzer. The basal respiration rate is measured in the first block. The respiration rate was analyzed with step-by-step injections of mitochondrial complex inhibitors such as 1.5 μM oligomycin A, 2 μM FCCP, and 0.5 μM rotenone–antimycin A following the manufacturer’s protocol.

### Steady-state metabolomics by LC–MS/MS

BMDMs were extracted for metabolites. The extracts were analyzed using Shimadzu LC Nexera X2 UHPLC coupled with a QTRAP 5500 LC MS/MS (AB Sciex). ACQUITY UPLC UPLC BEH Amide analytic column was used for chromatographic separation. The mobile phase was performed in buffer solution A (10 mM ammonium acetate in water, pH 8.8) and buffer solution B (10 mM ammonium acetate in acetonitrile/water (95/5), pH 8.2). The gradient elution was 95–61% buffer solution B in 7 min, 61–44% buffer solution B in 9 min, 61–27% buffer solution B in 9.2 min, and 27–95% buffer solution B in 10 min. Finally, The column was equilibrated with 95% buffer solution B 13C-nicotinic acid (Toronto Research Chemicals) was added as the internal standard. MultiQuant 3.0.2 software (AB Sciex) was used to integrate the extracted MRM peaks.

### Animals and modeling

Sirt3 wild-type and Sirt3 knock-out (C57BL/6*-Sirt3*^em1Smoc^, NM-KO-190368) mice were obtained from Shanghai Model Organisms (China). Animal experiment is conducted according to protocols approved by the Animal Committee of North Sichuan Medical College. Sirt3^+/+^ and Sirt3^−/−^ mice were pretreated with Viniferin (MCE, 120 mg/kg, i.p.) for 1 h before MSU crystals injection. The mice were divided into different groups based on different interventions.

To obtain a mouse model of acute gouty inflammation, 40μL of sterile saline was injected into the right hind paw as self-control, and 40 μL of MSU crystals (1 mg MSU crystals in 40 μL saline) was injected into the left hind paw as an experiment group. At the 24th hour, the thickness of the paws of the mice is analyzed by a digital vernier caliper. Then the mice were sacrificed under CO_2_ anesthesia before the paws were extracted. To obtain a mouse model of acute gouty peritonitis, MSU suspension (3 mg MSU crystals in 0.5 ml saline) were injected intraperitoneally, and the mice were sacrificed under CO_2_ anesthesia after 6 h to extract peritoneal fluid. After the peritoneal fluid was centrifuged, the supernatants were used for ELISA detection, and the precipitation was stained with the corresponding antibody for FCM detection.

### Histological studies and immunofluorescence

Mice paw tissues were embedded with paraffin and cut into the slice of 5 μm for hematoxylin–eosin (HE) staining and immunofluorescence.

### Statistical analysis

The values are expressed as mean ± standard difference (SD). Statistical analysis is performed using one-way ANOVA analysis of variance. All the statistical analyses are evaluated using the GraphPad Prism software (Version 6.0). It is considered statistically different if the *P* value is less than 0.05.

## Results

### The expression and deacetylation activity of Sirt3 were decreased in MSU crystal-induced inflammation

**Fig. 1:**
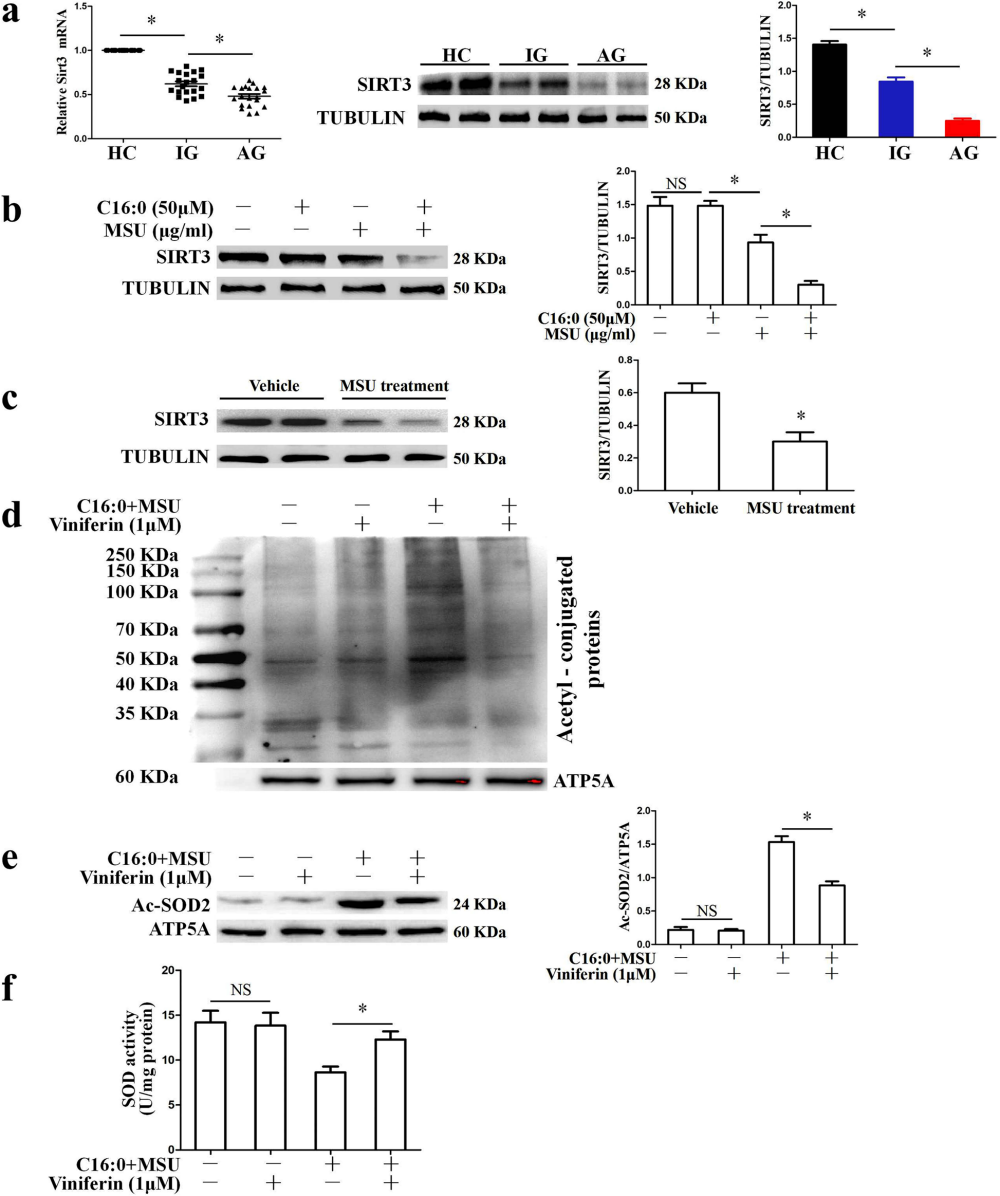
MSU crystals stimulation reduced the expression and deacetylation activity of Sirt3. **a** Sirt3 mRNA and protein levels were detected in PBMCs of health controls (HC), patients with acute gout (AG), and intermittent gout (IG). Sirt3 mRNA detection (HC, *n* = 24), (AG, *n* = 24), (IG, *n* = 24); GAPDH was used as an internal reference. Sirt3 protein detection (HC, *n* = 6), (AG, *n* = 6), (IG, *n* = 6), TUBULIN as an internal reference. **b** BMDMs were respectively stimulated with C16:0, MSU, or C16:0 + MSU for 12 h and Western blot was used to detect Sirt3 protein expression. **c** The protein levels of Sirt3 in the mouse paw treated with MSU crystals or vehicle. Data presented as mean ± SD (*n* = 5 mice per group). *P < 0.05. NS means no statistical difference. **d** Sirt3 agonists (Viniferin) inhibited the acetylation level of mitochondrial proteins in BMDMs treated with Viniferin (1 μM) and C16:0 + MSU crystals for 12 h. Acetyl-conjugated proteins in mitochondrial proteins were blotted with acetylated-lysine antibody. **e** Viniferin treatment decreased K68 lysine acetylation of mitochondrial SOD2 protein and mitochondrial proteins were analyzed by Western blot in BMDMs. **f** The activity of SOD in the mitochondrial proteins of BMDMs was detected with WST-8 method. Data are mean ± SD. Three independent repetitive experiments were conducted for each result. *P < 0.05. NS represents *P* > 0.05

It is reported that Sirt3 is involved in many inflammatory responses. The role of Sirt3 in MSU crystal-induced inflammation has not been clarified. In the study, the expression of Sirt3 in PBMCs of gout patients was measured. The mRNA and protein levels of Sirt3 in PBMCs of healthy volunteers were higher than those of gout patients (Fig. 1a). We also observed that patients with gout flare had significantly lower mRNA and protein levels of Sirt3 compared with intermittent gout (Fig. 1a). After BMDMs were treated with palmitic acid (C16:0), MSU crystals, and a combination of MSU crystals and palmitic acid for 12 h, C16:0 alone did not greatly affect Sirt3 protein expression, but C16:0 and MSU crystals synergistically lowered the SIRT3 protein level (Fig. 1b).

In the case of the MSU crystal-injected paws, the SIRT3 protein level remarkably decreased (Fig. 1c). SIRT3 is an important deacetylase of mitochondrial proteins [23]. Next, the acetylation level of the mitochondrial protein was detected. Stimulation of BMDMs by C16:0 + MSU markedly increased the overall acetylation of mitochondrial protein, whereas, a specific inducer/activator of Sirt3 [24], significantly reduced the acetylation level of the mitochondrial protein (Fig. 1d). Superoxide dismutase 2 (SOD2) is the most important target of Sirt3 deacetylation [25]. Viniferin inhibited the increase in the SOD2 acetylation level induced by C16:0 + MSU (Fig. 1e). The high deacetylation state of SOD2 can increase its activity. In addition, the activity of SOD in mitochondria was measured, and Viniferin reversed the decrease in SOD activity exposed to C16:0 + MSU (Fig. 1f). These data indicate that Sirt3-Sod2 pathway may be involved in the progression of gout inflammation.

### *Sirt3 deficiency enhanced mitochondrial ROS accumulation and NF-κB activation induced by C16:0* + *MSU*

**Fig. 2:**
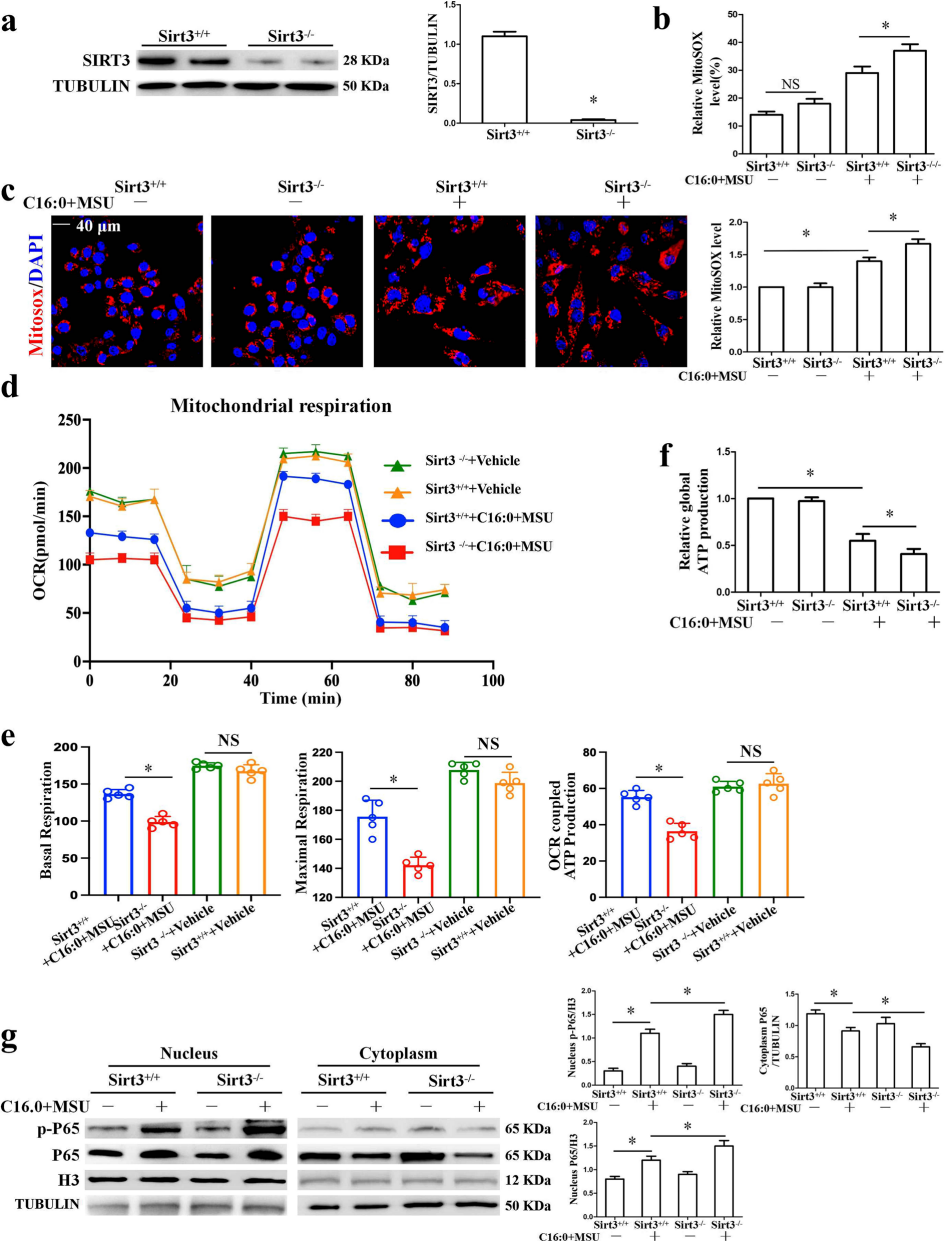
Sirt3 deficiency increased mtROS production and NF-κB activation in BMDMs treated with C16:0 + MSU. **a** Sirt3 protein levels of BMDMs from Sirt3^+/+^ and Sirt3^−/−^ mice. **b** The statistical analysis graph of mitochondrial ROS generation. **c** Mitochondrial ROS levels were measured by LCM image using a Mito-SOX probe. Red fluorescence represented mitochondrial ROS level. Blue shows nuclei staining with DAPI. Scale bar: 40 μm. **d** Mitochondrial respiration (oxygen consumption rate, OCR) was measured using Seahorse XFe24 Analyzer. **e** Quantification of basal respiration, maximal respiration, and OCR-coupled ATP production were analyzed (*n* = 5 biological replicates). **f** ATP Lite Luminescence Assay kit was used to detect global ATP production (*n* = 4 biological replicates). **g** Cytosolic and nuclear protein extracted from BMDMs were respectively reacted with anti-phosphorylated NF-κB P65 (p-P65) and anti-NF-κB P65 by western blotting. *P < 0.05. NS means no statistical difference

SOD2 is an important inhibitor of mitochondrial ROS. We examined whether Sirt3 deficiency promotes mitochondrial ROS aggregation induced by C16:0 + MSU. The Sirt3 protein expression was completely suppressed in Sirt3^−/−^ mice-derived BMDMs (Fig. 2a). Both FCM and LCM imaging analyses revealed that Sirt3 deficiency up-regulated mitochondrial ROS production induced by C16:0 + MSU. This is reflected in the increased fluorescence intensity of Mito-SOX (Sup Fig. 1a Fig. 2b and c). The effect of Sirt3 deficiency on the mitochondrial respiratory profile was detected. The data of the OCR indicated that Sirt3 deficiency diminished mitochondrial respiratory capacity in BMDMs treated with C16:0 + MSU, as evidenced by a decrease in basal respiration, the maximal respiratory capacity, and OCR-coupled ATP production (Fig. 2d and e). In addition, a decrease in global ATP production was observed in Sirt3^−/−^ BMDMs treated with C16:0 + MSU (Fig. 2f). These data support that Sirt3 plays an important role in maintaining the mitochondrial oxidative stress induced by C16:0 + MSU.

It is well demonstrated that the ROS/NF-κB signaling pathway plays an important role in MSU crystal-induced inflammation [26]. We investigated whether Sirt3 is involved in NF-κB activation. Then, cytosolic and nuclear fractions isolated from BMDMs were respectively probed for the phosphorylation levels of NF-κB subunit p65 (p-P65) protein through Western blot. These data indicate that Sirt3 deficiency elevated the protein levels of NF-κB p-P65 in the nucleus of BMDMs treated with C16:0 + MSU (Fig. 2g). The role of Sirt3 in NF-κB p65 nuclear translocation of BMDMs is further investigated by immunofluorescence assay. According to the data, in Sirt3 deficient BMDMs treated with C16:0 + MSU, the fluorescence intensity of NF-κB P65 in the nucleus was significantly increased (Sup Fig. 1b).

### *Sirt3 deficiency intensifies the inflammation induced by C16:0* + *MSU *in vitro

**Fig. 3:**
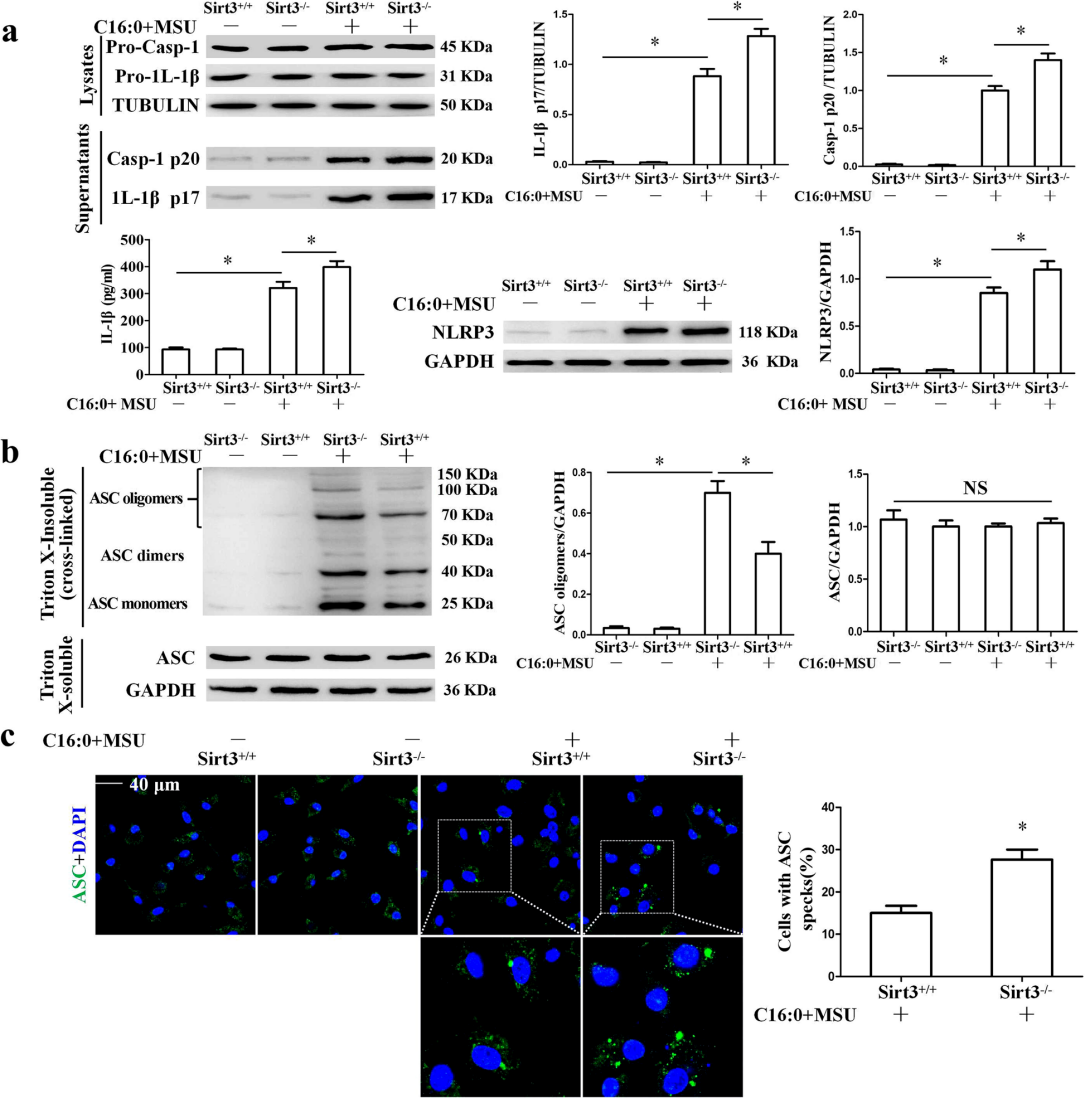
In BMDMs treated with C16:0 + MSU, Sirt3 deficiency accelerated NLRP3 inflammasome activation. **a** The protein levels of NLRP3, Caspase-1, and IL-1β in the cell lysates were evaluated by Western blot. Mature IL-1β (p17) and p20 fragments of Caspase-1 were detected in cell culture supernatants. **b** Total cell lysates were obtained in Triton X-100–containing buffer. Insoluble (pellet) fractions were cross-linked with DSS to capture ASC oligomers. The soluble and insoluble fractions were analyzed by Western blot with ASC antibody. **c** Representative LCM images of BMDMs stained with ASC antibody. DAPI staining nuclei. Scale bar, 40 μm. *P < 0.05. NS shows no statistical difference

The effect of Sirt3 deficiency on the pro-inflammation gene expression was further studied. Sirt3 deficiency accelerated the mRNA expression of pro-inflammatory cytokines (IL-1*β*, IL-6, and TNF-*α*) and pro-inflammatory enzymes (COX-2 and INOS) in BMDMs treated with C16:0 + MSU (Sup Fig. 2a). In accordance with qPCR data, Western blot analysis data also revealed that INOS and COX-2 protein levels were further increased in Sirt3 deficient BMDMs treated with C16:0 + MSU (Sup Fig. 2b). The NLRP3 inflammasome activation and IL-1β secretion play essential roles in the acute inflammation of gout. We wanted to examine the effect of Sirt3 deficiency on NLRP3 inflammasome activation. Sirt3 deficiency intensified Casp1 activation and pro-IL-1β processing to mature IL-1β (Fig. 3a). Consistent with its effect on Casp1 activation, Sirt3 deficiency further promoted ASC oligomerization (Fig. 3b) and ASC speck formation in BMDMs treated with C16:0 + MSU (Fig. 3c). Overall, these findings indicate that Sirt3 deficiency accelerates the inflammation induced by C16:0 + MSU.

### *Sirt3 deficiency accelerated Acod1 expression in the inflammation induced by C16:0* + *MSU*

**Fig. 4:**
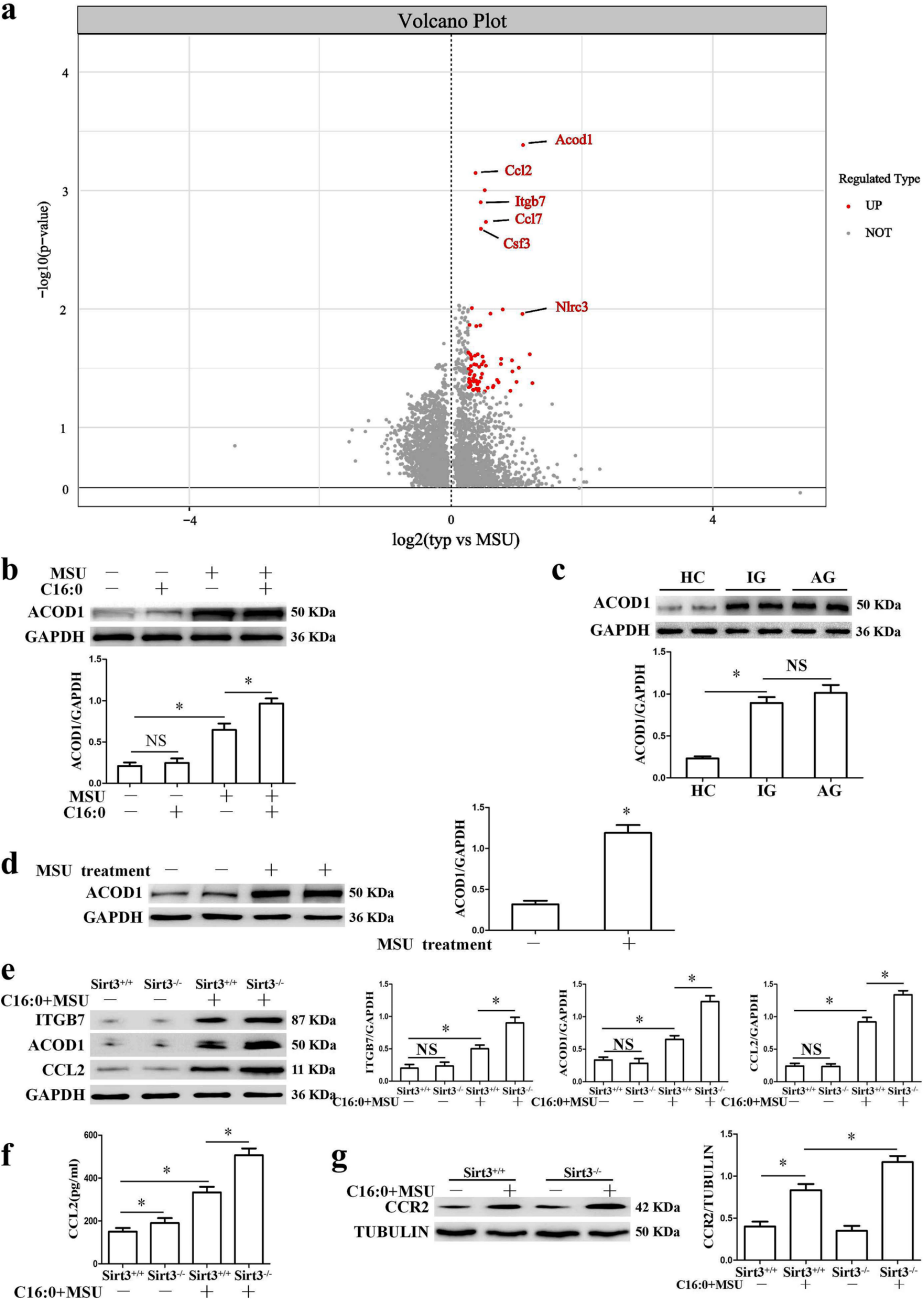
Sirt3 deficiency resulted in further up-regulation of Acod1 expression in BMDMs treated with C16:0 + MSU. **a** The volcano plot is used to indicate the up-regulated (in red) ribonucleic acids in the Sirt3^−/−^ BMDMs treated with C16:0 + MSU. Red spots represent − log10(P) values of ≥ 2. **b** BMDMs were stimulated with C16:0, MSU, or C16:0 + MSU for 12 h. Western blot was used to detect Acod1 protein levels. **c** Acod1 protein levels in PBMCs from HC, AG and IG. **d** The protein levels of ACOD1 in the mouse paw injected with MSU crystals or vehicle. **e** ITGB7, ACOD1, and CCL2 protein expression was up-regulated in Sirt3 deficient BMDMs in response to C16:0 + MSU. **f** and** g** CCL2 secretion and CCR2 protein expression were detected in Sirt3 deficient BMDMs treated with C16:0 + MSU. *P < 0.05. NS shows no statistical difference

In order to understand the molecular mechanism of Sirt3 modulating the inflammation induced by MSU crystals, RNA sequencing, and bio-informatics analysis were performed to detect gene expression profiles in Sirt3^+/+^ and Sirt3^−/−^ BMDMs treated with C16:0 + MSU. Hierarchal clustering divides different expression genes (DEGs) into two different clustering in Sirt3^−/−^ and Sirt3^+/+^ BMDMs treated by C16:0 + MSU. This means that sequencing data is very reliable (Sup Fig. 3a). Compared with Sirt3^+/+^ BMDMs treated with C16:0 + MSU, a total of 31 genes presented significant up-regulation in Sirt3^−/−^ BMDMs treated with C16:0 + MSU (Table 5). The DEGs were visualized with a volcano plot (Fig. 4a). The Kyoto Encyclopedia of Genes and Genomes(KEGG) enrichment indicates that Sirt3 contributes significantly to the expression patterns of the IL-17 signaling pathway (including Ccl2, Ccl7, and Csf3) (Sup Fig. 3b). Furthermore, RT-qPCR was used to validate RNA sequencing and bio-informatics analysis data including some well-known genes associated with inflammation, such as Acod1 (Irg1), Ccl2, Nlrc3 (Nlrp3), Itgb7, and Ccl7. The RT-qPCR data indicated that Sirt3 deficiency could accelerate the expression of these genes induced by C16:0 + MSU (Sup Fig. 3c). On the other hand, Viniferin inhibited the mRNA levels of these genes induced by C16:0 + MSU crystals (Sup Fig. 4a). We also analyzed the DNA motifs of these five differentially expressed genes. Motif enrichment analysis was performed using RcisTarget (version 1.18.2). The transcription factors (TF)-binding motifs of the upstream 10 kb sequence of the selected differentially expressed genes were analyzed using the database it provided. The data was shown in Sup Fig. 4b.

**Table 5:** The differentially expressed genes between Sirt3^+/+^ and Sirt3^−/−^ BMDMs treated with C16:0 + MSU

Gene	ID	Log2fold change	*p*-value
Dab2	ENSMUSG00000022150	1.0133	1.30E − 04
Plekho1	ENSMUSG00000015745	1.0159	1.04E − 15
Fblim1	ENSMUSG00000006219	1.0201	1.23E − 11
Urah	ENSMUSG00000025481	1.0279	1.26E − 13
Anpep	ENSMUSG00000039062	1.041	1.10E − 10
Il1rn	ENSMUSG00000026981	1.0428	1.64E − 17
Nlrc3	ENSMUSG00000049871	1.0774	3.22E − 04
Lilr4b	ENSMUSG00000112023	1.1133	4.35E − 11
Sdc3	ENSMUSG00000025743	1.1235	2.24E − 10
Pmp22	ENSMUSG00000018217	1.158	6.12E − 07
Ccl2	ENSMUSG00000035385	1.1706	1.47E − 13
Acod1	ENSMUSG00000022126	1.1799	1.49E − 32
Ccrl2	ENSMUSG00000043953	1.1944	2.62E − 04
Tbc1d9	ENSMUSG00000031709	1.2033	2.27E − 06
Hmga2	ENSMUSG00000056758	1.2083	2.50E − 06
Dhrs9	ENSMUSG00000027068	1.2452	5.43E − 06
Ptgir	ENSMUSG00000043017	1.2452	3.24E − 05
Fam20c	ENSMUSG00000025854	1.2715	2.78E − 09
Fabp4	ENSMUSG00000062515	1.2876	1.84E − 04
Parvb	ENSMUSG00000022438	1.2959	3.82E − 07
Slamf7	ENSMUSG00000038179	1.3104	3.65E − 06
Klf2	ENSMUSG00000055148	1.411	6.37E − 05
Ccl7	ENSMUSG00000035373	1.4653	9.76E − 10
Csf3	ENSMUSG00000038067	1.5185	6.58E − 08
Emp1	ENSMUSG00000030208	1.5515	1.44E − 17
Slc6a8	ENSMUSG00000019558	1.7697	3.88E − 19
Gpnmb	ENSMUSG00000029816	1.8338	5.49E − 20
Ptpn14	ENSMUSG00000026604	2.1787	6.70E − 05
Itgb7	ENSMUSG00000001281	2.2451	3.26E − 16
Alb	ENSMUSG00000029368	3.6745	5.61E − 04

Among these up-regulated genes, we were particularly interested in Acod1 (Irg1), which has the most significant statistical difference. It has been reported that the mRNA level is remarkably elevated in MSU crystal-induced inflammation [27]. BMDMs were stimulated with C16:0 and MSU crystals for 12 h, and the Acod1 protein expression was detected. Interestingly, the effects of C16:0 on the Acod1 protein expression were very small, and C16:0 plus MSU crystals markedly accelerated the Acod1 protein level (Fig. 4b). The Acod1 protein levels in PBMCs of healthy controls were lower than those of gout patients (Fig. 4c). There was no significant difference in Acod1 protein levels between AG and IG (Fig. 4c). Western blot analysis revealed that Acod1 protein levels significantly increased in mouse paw injected with MSU crystals (Fig. 4d).

In addition, the effect of SIRT3 deficiency on ACOD1, ITGB7, and CCL2 protein levels was detected. Western blot data indicated that Sirt3 deficiency accelerated ACOD1, ITGB7, and CCL2 protein expression in BMDMs treated with C16:0 + MSU (Fig. 4e). Consistent with RT-qPCR and Western blot data, ELISA analysis showed that Sirt3 deficiency promoted CCL2 secretion induced by C16:0 + MSU (Fig. 4f). CCR2 is the major receptor of CCL2. In BMDMs treated with C16:0 + MSU, it was also observed that Sirt3 deficiency increased the CCR2 expression (Fig. 4g). The effect of Viniferin on several key proteins associated with C16:0 + MSU-induced inflammation was detected. Viniferin treatment reduced ITGB7, CCR2, ACOD1, NLRP3 protein expression and CCL2 secretion (Sup Fig. 4c and d).

We wanted to know whether the Sirt3-Sod2 pathway inhibited Acod1 expression by ROS/NF-κB signaling. Mito-TEMPO, a mitochondrial ROS scavenger, effectively inhibits the up-regulation of Acod1 mRNA and protein expression stimulated by C16:0 + MSU (Sup Fig. 5a). Mito-TEMPO treatment moderated the up-regulation of Acod1 expression in Sirt3^−/−^ BMDMs treated with C16:0 + MSU (Sup Fig. 5b). HY-133987 (the inhibitor of NF-κB) treatment suppressed the elevation of Acod1 expression induced by C16:0 + MSU in Sirt3^−/−^ BMDMs (Sup Fig. 5c). All these data imply that the Sirt3-Sod2 pathway may decrease C16:0 + MSU crystal-induced Acod1 expression by inhibiting mtROS levels and NF-κB activation.

### *Acod1 knockdown reduced mtROS production and injury induced by C16:0* + *MSU*

**Fig. 5:**
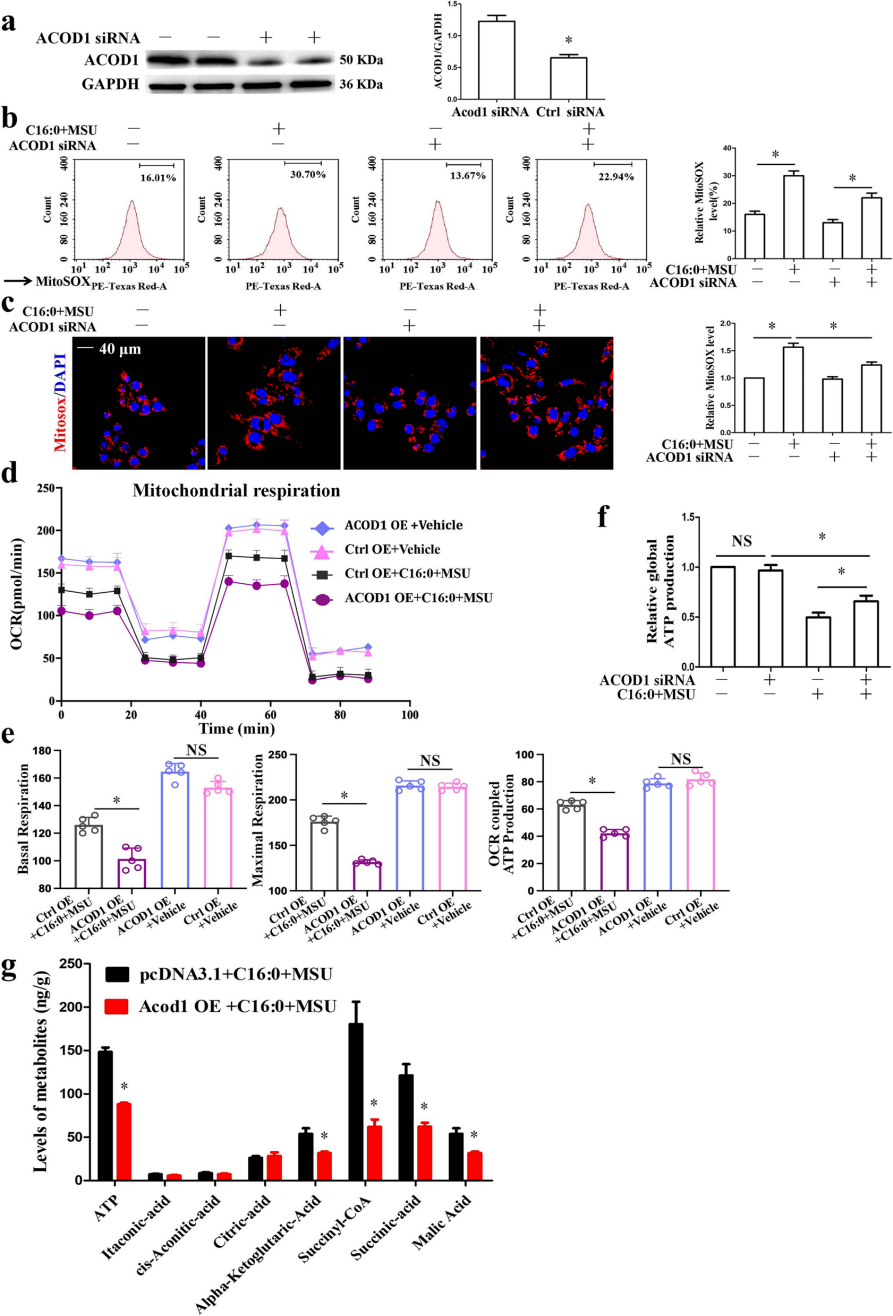
Acod1 regulated mitochondrial ROS levels, the activation of NF-κB, and the intermediate metabolite production in the TCA cycle. **a** BMDMs were transfected with Acod1 siRNA or Ctrl siRNA for 36 h, Western blot was used to detect ACOD1 protein level. **b** Mitochondrial ROS production was measured by FCM using Mito-SOX probes. The relative level of Mito-SOX was on the right. **c** The mitochondrial ROS level was determined by LCM images using the Mito-SOX probe. Red fluorescence represented mitochondrial ROS level. The relative level of Mito-SOX was on the right. **d** BMDMs were over-expressed Acod1 and treated with C16:0 + MSU. Mitochondrial respiration (oxygen consumption rate, OCR) was measured. **e** Quantification of basal respiration, maximal respiration, and OCR-coupled ATP production in mitochondrial respiration (*n* = 5 biological replicates). **f** The global ATP production was detected. **g** Relative levels of metabolites, assessed by LC–MS/MS analysis, in BMDMs transfected pcDNA3.1 empty plasmid (Ctrl OE) and inserted Acod1 ORF into pcDNA3.1 plasmid (ACOD1 OE) for 36 h, and then treated with C16:0 + MSU for 12 h. *n* = 4 independent samples. NS represents *P* > 0.05

It has been reported that Acod1 can accelerate ROS levels in the Zebrafish model of acute gouty inflammation [8]. As shown in Fig. 5a, Acod1 expression was partially inhibited in BMDMs transfected with targeted Acod1 siRNA. In this study, we tried to investigate whether Acod1 knockdown could affect mtROS levels induced by C16:0 + MSU. Acod1 knockdown obviously inhibited mtROS production stimulated by C16:0 + MSU (Fig. 5b and c). We also investigated the effect of Acod1 over-expression on mitochondrial respiration. In Acod1 over-expression BMDMs treated with C16:0 + MSU, basal respiration, maximal respiratory capacity, and OCR-coupled ATP production were decreased (Fig. 5d and e). Acod1 knockdown partially restored the C16:0 + MSU crystal-induced reduction in global ATP production (Fig. 5f). These data support that Acod1 knockdown can significantly improve mitochondrial function in BMDMs exposed to C16:0 + MSU. Next, targeted steady-state metabolomics was used to investigate how Acod1 over-expression influences energy metabolism (Fig. 5g).

The TCA cycle is the core metabolic pathway that produces ATP. As shown in Fig. 5g, Acod1 over-expression has significant effects on the levels of some metabolites associated with the TCA cycle. ACOD1 catalyzes the production of itaconic acid through cis-Aconitic Acid decarboxylation. But in BMDMs treated with C16:0 + MSU, Acod1 over-expression had little effect on the levels of itaconic acid and cis-aconitic acid (Fig. 5g). No significant change in Citric Acid concentration was observed (Fig. 5g). More importantly, the data showed that Acod1 over-expression significantly reduced the level of TCA cycle intermediates (α-ketoglutaric acid decreased to 41.5%, succinyl-CoA decreased to 66.1%, succinic acid decreased to 48.7% and malic acid decreased to 43.3%) (Fig. 5g). All these data indicated that Acod1 over-expression might induce mitochondrial oxidative damage and disrupt mitochondrial metabolism.

### Sirt3 and Acod1 regulated BMDMs migration

**Fig. 6:**
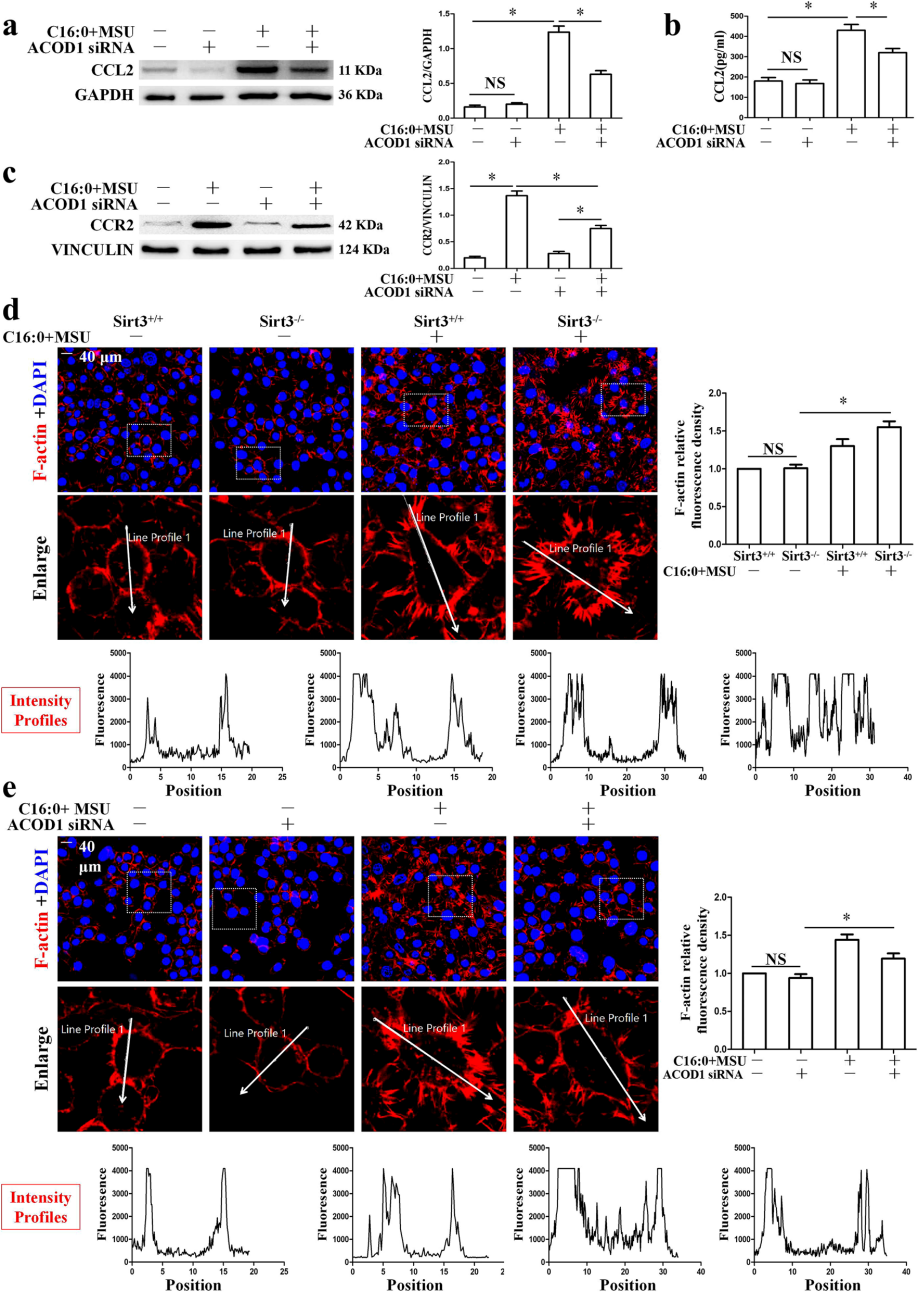
Acod1 knockdown suppressed CCL2 and CCR2 expression. Sirt3 deficiency or Acod1 knockdown affected BMDM migration-induced C16:0 + MSU. **a**–**c** The CCL2 and CCR2 expression level was detected in BMDMs transfected siRNA for 36 h and then treated with C16:0 + MSU for 12 h. **d** and **e** BMDMs from Sirt3^+/+^ and Sirt3.^−/−^ mice treated with C16:0 + MSU or BMDMs transfected with siRNA and then treated with C16:0 + MSU. Rhodamine phalloidin-labeled F-actin (red) and DAPI-labeled nucleus (blue) were analyzed by confocal microscopy. Representative cells in each group surrounded in the white frames were enlarged. Bottom panel shows line scans (along the straight white line) of fluorescence intensities with F-actin localization. A histogram chart of relative fluorescence intensity was on the right. Scar bar, 40 μm. *P < 0.05. NS indicates *P* > 0.05

Since Sirt3 inhibited Acod1 and Ccl2 expression, we wanted to further explore whether Acod1 knockdown could suppress Ccl2 and Ccr2 expression. Acod1 knockdown markedly blocked the elevation of Ccl2 and its major receptor Ccr2 expression induced by C16:0 + MSU (Fig. 6a–c). Ccl2 and Ccr2 are important regulators of macrophage recruitment. We investigated the effects of Sirt3 over-expression and Acod1 knockdown on the migration ability of BMDMs treated with C16:0 + MSU. The expression of F-actin is closely related to the movement and migration of cells [28]. Phalloidin staining data revealed that the formation of F-actin was increased in Sirt3 deficient BMDMs treated with C16:0 + MSU (Fig. 6d). While the formation of F-actin was greatly repressed in Acod1 knockdown BMDMs treated with C16:0 + MSU (Fig. 6e). These findings indicate that in the gout arthritis, Sirt3 and Acod1 may be involved in recruitment of inflammatory cells to inflammatory site by regulating cell migration.

### *Acod1 knockdown improved mitochondrial fragmentation induced by C16:0* + *MSU*

**Fig. 7:**
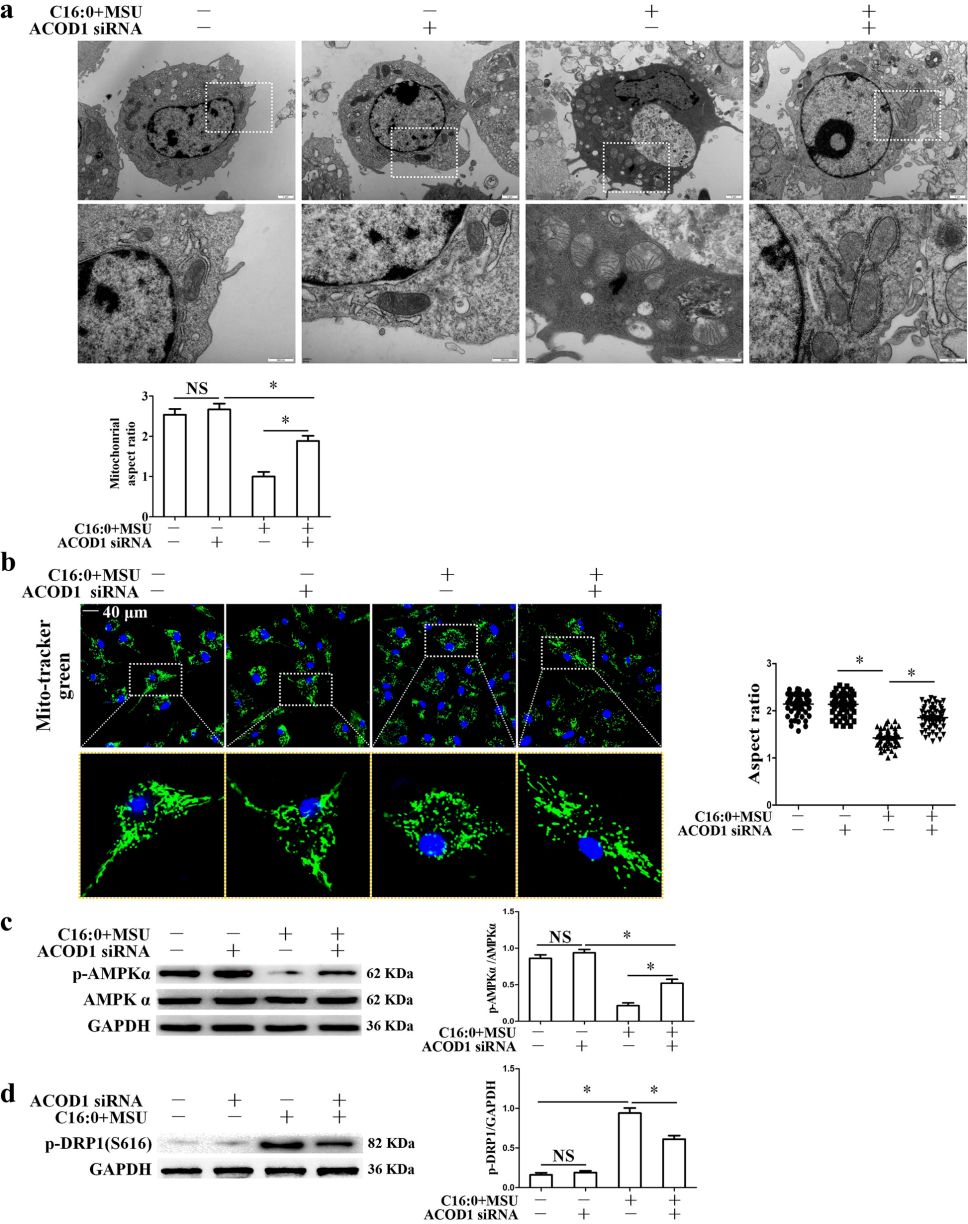
Acod1 knockdown improved mitochondrial morphology, promoted AMPK activation, and inhibited Drp1 protein expression. **a** Mitochondrial structure was examined by TEM in the BMDMs. Statistical analysis of the mitochondrial aspect ratio is shown at the bottom. Mitochondria were analyzed in at least 10 fields. **b** Representative LCM images of Mito-Tracker green probe in live BMDMs mitochondrial imaging. Representative cells in each group surrounded in the white frames were enlarged. The box and whisker plot of the quantified mitochondrial aspect ratio was on the right; DAPI stains nuclei. Scale bar, 40 μm. Mitochondria were analyzed in at least 10 fields. **c**, **d** Western blot was used to detect AMPKα, phosphorylation of AMPKα, and phosphorylation of Drp1. *P < 0.05. NS represents no statistical difference

Mitochondrial ROS over-production is closely related to fragmentation. Acod1 promoted mitochondrial ROS generation. We sought to understand whether Acod1 knockdown could contributes to the improvement of mitochondrial structure in BMDMs treated with C16:0 + MSU. Ultrastructural analysis of the transmission electron microscopy (TEM) revealed that there was an increased tendency of mitochondrial fragmentation in BMDMS treated with C16:0 + MSU. In particular, some small and round mitochondria appeared and were parallel to the disruption of the mitochondrial network (Fig. 7a). The mitochondrial morphology in BMDMs stained with Mito-Tracker Green was evaluated by laser confocal microscopy (LCM). We also analyzed the mitochondrial aspect ratio (AR) values, which reflect the mitochondrial size. As shown in Fig. 7a and b, Acod1 knockdown significantly reversed the decrease of mitochondrial AR value induced by C16:0 + MSU, indicating an improvement in mitochondrial fragmentation.

**Fig. 8:**
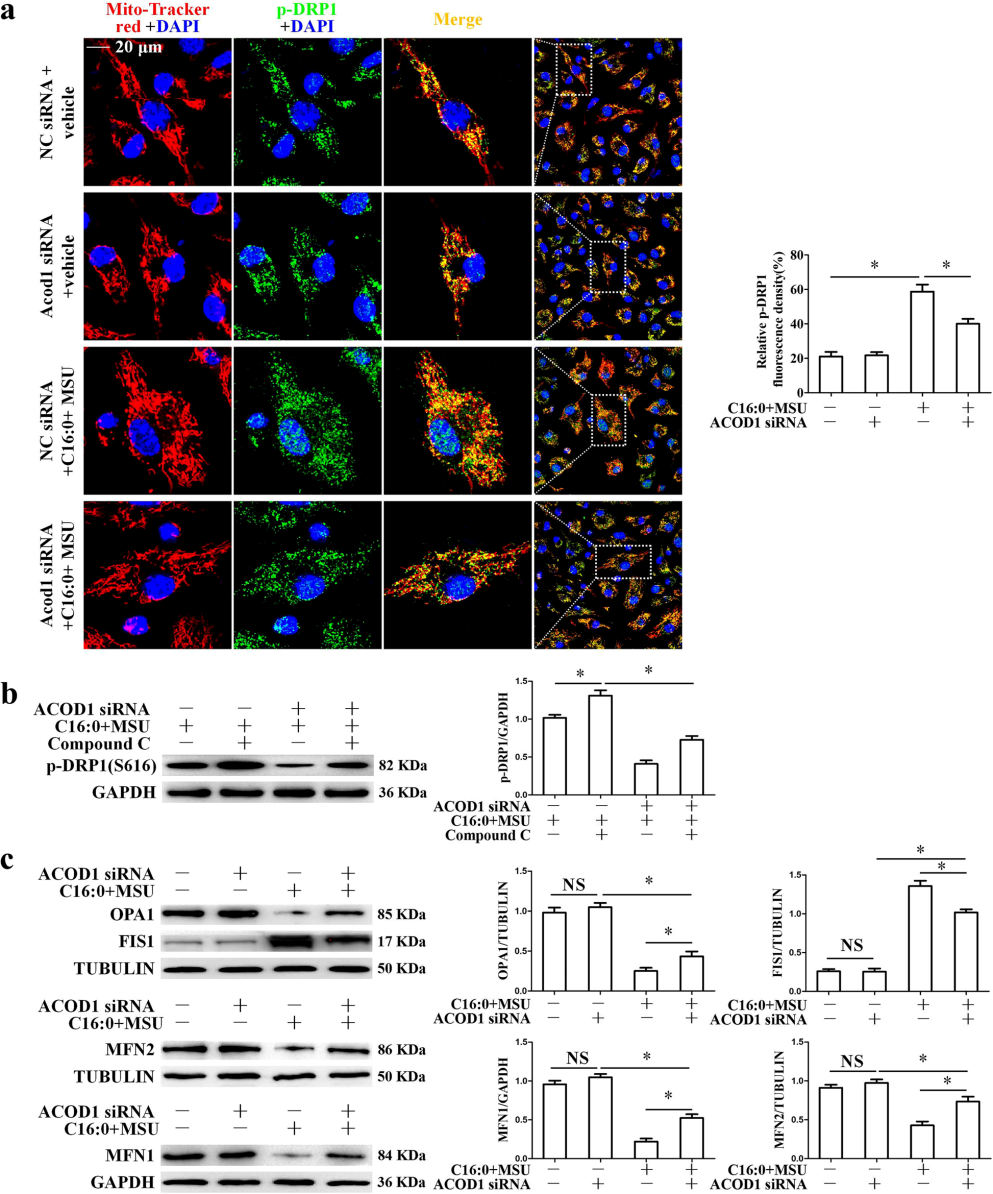
Acod1 knockdown decreased Drp1 localization in mitochondria and FIS1 protein levels and promoted OPA1, MFN1, and MFN2 protein levels. Compound C reversed the effect of Acod1 knockdown on p-Drp1 protein levels. **a** Representative LCM images of BMDMs co-stained with Mito-Tracker Red probe and p-DRP1 Ab. DAPI stains nuclei. Scale bar, 20 μm. Representative cells in each group surrounded in the white frames were enlarged. A histogram chart of relative fluorescence intensity was on the right. **b** and **c** The protein levels of p-Drp1, OPA1, FIS1, MFN1, and MFN2 were detected using Western blot. *P < 0.05. NS means no statistical difference

MSU crystals suppress the phosphorylation of AMPKα in BMDMs [29], and Sirt3 over-expression can suppress mitochondrial fission by normalizing AMPK-DRP1 pathways [30]. Next, we attempted to investigate whether Acod1 could affect phosphorylation (activation) of AMPKα Thr172 in BMDMs treated with C16:0 + MSU. Acod1 knockdown repressed the reduction of phosphorylation of AMPKα induced by C16:0 + MSU (Fig. 7c). We sought to monitor the effect of Acod1 knockdown on Drp1 activity. Acod1 knockdown decreased the phosphorylation level of Drp1 (Ser616, p-DRP1) induced by C16:0 + MSU (Fig. 7d). The immunofluorescence assay revealed that mitochondrial recruitment of p-Drp1 in BMDMs exposed to C16:0 + MSU was significantly blocked by Acod1 knockdown (Fig. 8a). We also found that compound C (AMPK inhibitor) abolished the role of Acod1 knockdown in inhibiting the up-regulation of p-Drp1 induced by C16:0 + MSU (Fig. 8b). Previous study has revealed that Sirt3 deficiency affects the expression of mitochondrial fission–fusion protein [31]. We also analyzed the effect of Acod1 knockdown on the expression of other mitochondrial fission–fusion proteins. This was accompanied by a decrease in the expression of mitochondrial fission protein (FIS1) and an increase of mitochondrial fusion proteins (OPA1, MFN1, and MFN2) levels (Fig. 8c), suggesting that Acod1 knockdown inhibited mitochondrial fission and promoted mitochondrial fusion. Taken together, these data suggest that Acod1 is involved in the mitochondrial dynamic remodeling of BMDMs exposed to C16:0 + MSU.

### *Sirt3 deficiency exacerbated MSU crystal-induced inflammation *in vivo

**Fig. 9:**
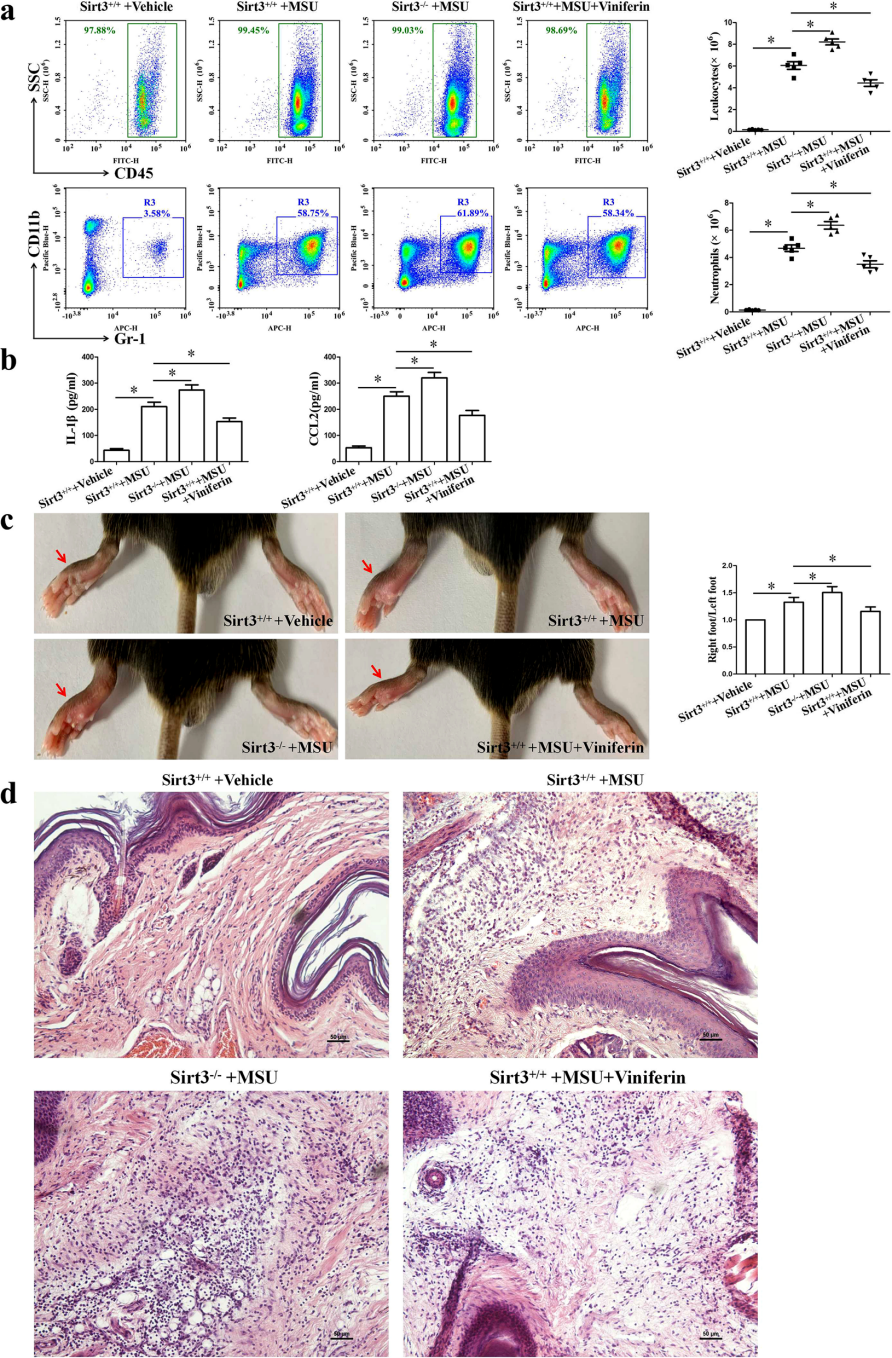
Sirt3 deficiency exacerbated MSU crystal-induced inflammation in vivo. **a** Representative plots of migrated leukocytes (CD45^+^) and neutrophils (CD11b^+^Gr-1.^+^) in the peritoneal fluid were detected by FCM. The number of migrated leukocytes and neutrophils were quantified and compared among the groups on the right. **b** The levels of IL-1β and CCL2 in the peritoneal fluid were detected by ELISA. **c** Paw swelling index. **d** Representative microscopic images of mouse paw sections stained with HE (magnification 20 × 10). *n* = 5 mice for each group. *P < 0.05

Based on the observed findings of Sirt3 on NF-kB p65 nuclear translocation and NLRP3 inflammasome activation in vitro, we tested whether Sirt3 deficiency aggravated MSU crystal-induced peritonitis, or whether Viniferin is protective against MSU crystal-induced peritonitis in mice. The number of leukocytes and neutrophils in the peritoneal fluid were evaluated by leukocyte marker CD45 and the neutrophil marker Gr-1 staining for cells. As shown in Fig. 9a, compared to Sirt3^+/+^ mice injected with MSU crystals, Sirt3^−/−^ mice injected with MSU crystals showed extensive accumulation of leukocytes and neutrophils in peritoneal lavage fluid, along with an increase in the secretion of IL-1β and CCL2 (Fig. 9b). We found that Viniferin attenuated MSU crystal-induced peritonitis, as evidenced by a lower leukocytes and neutrophils flux and decreased IL-1β and CCL2 production in peritoneal lavage fluid, compared to Sirt3^+/+^ mice injected with MSU crystals (Fig. 9a and b).

**Fig. 10:**
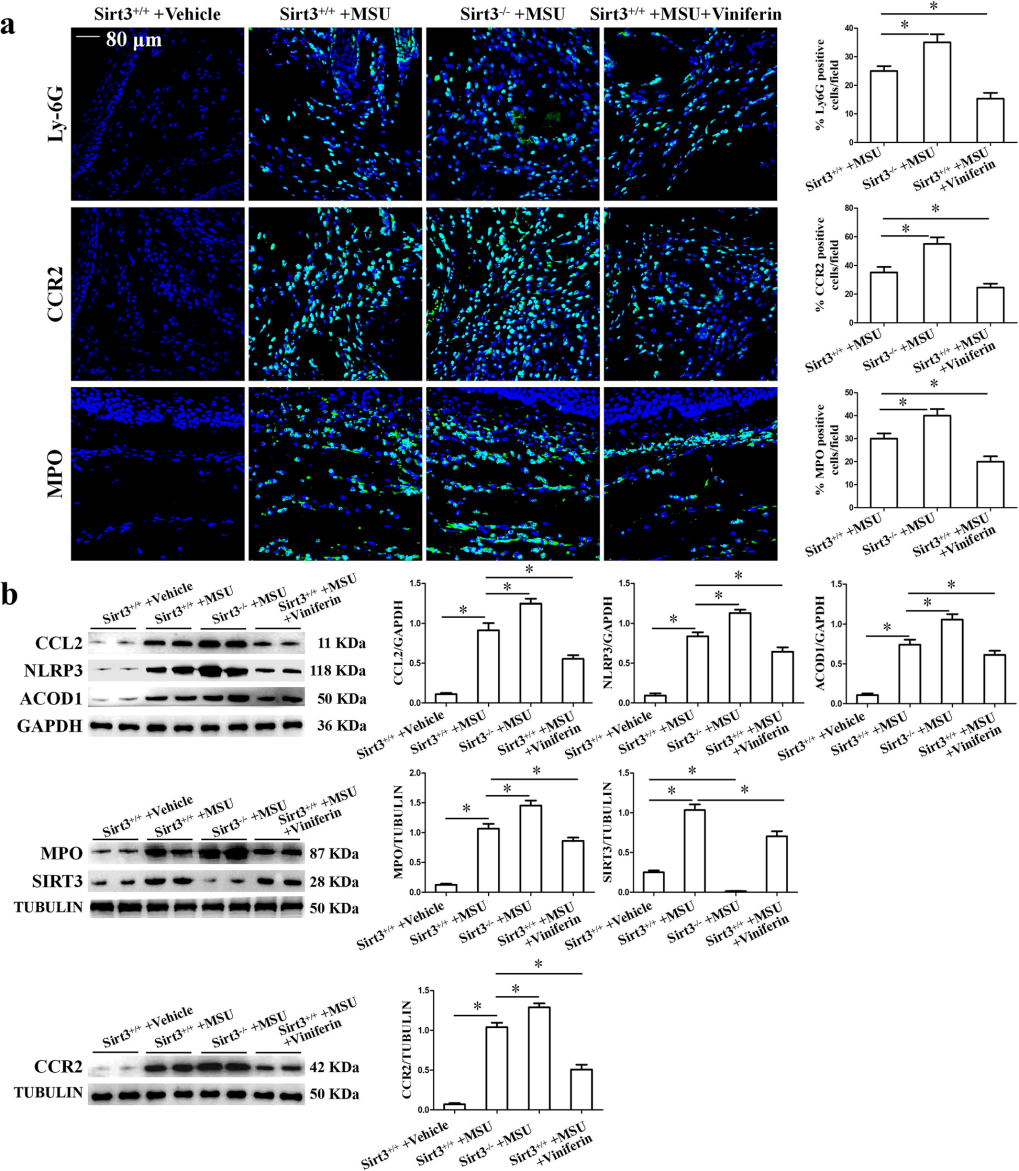
Sirt3 deficiency aggravated MSU crystal-induced inflammatory cell infiltration and inflammation-related protein expression in the paw of mice. **a** The distribution of Ly-6G, CCR2, and MPO-positive cells in mouse paw tissue sections was detected by immunofluorescence assay. Blue shows nuclei stained with DAPI. Scale bar, 80 μm. *n* = 5 mice per group. Ten to 12 images per mouse were analyzed. **b** Representative Western blot and statistical analysis of protein levels of CCL2, NLRP3, ACOD1, MPO, SIRT3, and CCR2 in mouse paw tissue homogenates. Data presented as mean ± SD (*n* = 5 mice per group). *P < 0.05

We also examined the effects of Sirt3 deficiency or Viniferin treatment on the swelling of MSU crystal-induced paw and invasion of inflammatory cells. In comparison with Sir3^+/+^ mice injected with MSU crystals, Sirt3^−/−^ mice injected with MSU crystals exhibited higher paw swelling index (Fig. 9c). Viniferin treatment significantly decreased the swelling of mice paw of Sirt3^+/+^ mice induced by MSU crystals (Fig. 9c). H&E staining data showed immune cell distribution consistent with paw swelling index (Fig. 9d). Immunofluorescence staining showed that Sirt3 deficiency promoted the distribution of MPO, Ly6G, and CCR2-positive cells in MSU crystal-injected paw tissue sections (Fig. 10a). Meanwhile, Sirt3 deficiency promoted the expression of ACOD1, MPO, NLRP3, CCL2, and CCR2 proteins in MSU crystal-injected paw tissue (Fig. 10b). Viniferin effectively diminished MSU crystal-induced inflammatory indexes in Sirt3^+/+^ mice, as evidenced by a lower paw swelling and reduced accumulation of leukocytes (Fig. 9c, d). Western blot analysis of paw tissue homogenates revealed that Viniferin significantly prevented the reduction of Sirt3 expression in Sirt3^+/+^ mice injected with MSU injection (Fig. 10b). Viniferin also diminished MSU crystal-induced ACOD1, MPO, NLRP3, CCL2, and CCR2 protein expression in Sirt3^+/+^ mice paw tissue homogenates (Fig. 10b). These anti-inflammatory effects of Viniferin were abrogated in Sirt3^−/−^ mice subjected to MSU crystal-induced peritonitis (Sup Fig. 6a and b). These findings suggest that SIRT3 is an important and potential therapeutic target for inflammation induced by MSU crystals.

## Discussion

Long-term hyperuricemia induces the deposition of MSU crystals into multiple tissues and organs. Immune cells such as monocytes/macrophages, neutrophils, and mast cells “recognize” MSU crystals in the joints and trigger immune response. Among these immune cells, resident macrophages play an important role in the initiation of acute gout [32]. The present study investigated the role and molecular mechanism of Sirt3 in MSU crystal-induced inflammation.

The Sirt3 expression is decreased in the PBMCs of gout patients. C16:0 and MSU crystals synergistically suppressed the expression of Sirt3 in macrophages. Sirt3 is a major regulator of deacetylation for mitochondrial proteins [23]. C16:0 + MSU crystals stimulation increased the acetylation level of mitochondrial proteins. C16:0 + MSU crystals stimulation not only inhibits the expression of Sirt3, but also reduces the activity of Sirt3. Sirt3 agonists significantly inhibited the acetylation level of mitochondrial proteins induced by C16:0 and MSU crystals. More importantly, Sirt3 agonists blocked the SOD2 acetylation level, which is the most important target of Sirt3 in mitochondria [25]. It has been proved that the Sirt3-Sod2 pathway can inhibit mitochondrial oxidative stress and NLRP3 inflammasome activation [21, 33]. The current study indicated that Sirt3 deficiency promoted the ROS/NF-κB signaling and NLRP3 inflammasome activation in response to C16:0 + MSU. Sirt3 deficiency accelerates the invasion of immune cell in vivo. Viniferin treatment markedly improved the inflammation induced by MSU crystals in vivo and in vitro. All of these data indicate that the Sirt3-Sod2 pathway plays a protective role in MSU crystal-induced inflammation.

The molecular mechanism of Sirt3 in C16:0 + MSU-induced inflammation was further explored. RNA sequencing and bio-informatics analysis revealed that Sirt3 deficiency significantly promoted the mRNA expression of Acod1, and Acod1 was identified as one of the most highly up-regulated genes in a mice air pouch model of acute gouty inflammation, indicating a potential pathological role of Acod1 in gout [27]. In previous studies, macrophages have been shown to promote the production of mitochondrial ROS in the inflammation induced by C16:0 and MSU crystals, depending on the expression of Acod1 [17]. However, the relationship between Sirt3 and Acod1 has not been reported. The present study reveals that the Sirt3-Sod2 pathway conducts negative regulatory Acod1 expression through ROS/NF-kB signal in MSU crystal-induced inflammation.

The role of ACOD1-dependent itaconic acid is well-known in disease [34–38], the regulation and function of ACOD1 independent of itaconic acid remains poorly understood. In the present study, metabolomic data indicated that Acod1 over-expression did not affect the production of itaconic acid in BMDMs treated with C16:0 + MSU. Acod1 over-expression significantly reduced the levels of important intermediate metabolites in the TCA cycle including malic acid, succinic acid, succinyl-CoA, and α-ketoglutaric acid in BMDMs exposed to C16:0 + MSU. Recent studies have shown that Sirt3 can promote α-ketoglutaric acid production in macrophages [39]. These data reflect that Sirt3 may increase α-ketoglutaric acid levels by inhibiting Acod1 expression. The accumulation of ACOD1 results in a powerful pro-inflammatory response independent of itaconic acid [40]. Consistent with this study, our data suggest that Acod1 is capable of exacerbating C16:0 + MSU-induced inflammation independent of itaconic acid.

We investigated whether Acod1 knockdown affected mtROS, mitochondrial respiration, and mitochondrial dynamics. Acod1 knockdown could restore cellular energy metabolism in response to C16:0 + MSU. As a dynamic organelle, mitochondria function is tightly associated with the balance of the fusion/fission process. Our findings indicate that Acod1 knockdown inhibits mitochondrial fragmentation and improves mitochondrial dynamic equilibrium. Previous study has indicated that Sirt3 can activate AMPK [41]. AMPK plays an important role in maintaining the integrity of the mitochondrial structure by limiting the translocation of Drp1 to mitochondria [42]. The study further unveiled that Acod1 knockdown induced AMPK activation, as reflecting elevated p-AMPK levels. Compound C treatment reversed the effect of Acod1 knockdown on AMPK and Drp1 activity, suggesting that Acod1 knockdown may moderate the effect of MSU on mitochondrial dynamics remodeling by promoting AMPK activity. These data support that Acod1 knockdown inhibits mitochondrial dysfunction induced by C16:0 + MSU.

Sirt3 and Acod1 also affect the expression of CCL2 (Mcp-1). CCL2 is a chemokine and is associated with the recruitment and migration of monocytes/macrophages. Uric acid promotes Mcp-1 production, which is an important component of the immune response to hyperuricemia and gout [43, 44]. Several circulating miRNAs associated with hyperuricemia and gout affect CCl-2 expression [45]. Injection of joints with IL-1β has been shown to be a trigger for increased CCL2 production [46]. Recently, it was found that JNK activation correlates with serum CCL2 release in an animal model induced by MSU crystals [47]. In the current study, Sirt3 and Acod1 could affect CCl2 and its major receptor CCR2 expression. Sirt3 deficiency increased macrophage migration. On the other hand, Acod1 knockdown reduced macrophage migration in response to C16:0 and MSU crystals. In vivo, Sirt3 deficiency significantly increased inflammatory cell infiltration. These data suggest that Sirt3 may influence the migration of inflammatory cells through CCL2.

## Conclusions

Sirt3 plays an important anti-inflammatory role in inflammation induced by MSU crystals. The Sirt3-Sod2 pathway negatively regulates Acod1 expression at the transcriptional level through ROS/NF-kB signaling. Acod1 exerts a pro-inflammatory effect on inflammation induced by MSU crystals, independent of the effect on Itaconic acid metabolism.

## Supplementary Information

**Additional file 1:**  **Sup Fig. 1. **Sirt3 deficiency increased mitochondrial ROS production and nuclear localization of NF-kB P65in BMDMs treated with FAs + MSU crystals.** a **Flow analysis plotsusing FCM to detect the production of mitochondrial ROS after MitoSOX probe staining of BMDMs. **b **The percentages of NF-κB p65 in the BMDMsnucleus were quantified through immune-fluorescence assay. Blue shows nuclei staining with DAPI. Scale bar:40 μm. *P < 0.05. **Sup Fig. 2. **Sirt3 deficiency accelerated the expression of inflammation associated gene in BMDMs treated with C16:0 + MSU. **a **Quantitative PCR analysis was used to detect IL-1*β*,IL-6, TNF*α*, COX-2, and iNOS mRNA expression. **b **The protein levels of COX-2 and INOS. **Sup Fig. 3. **Expression analysis of differentially expressed genes (DEGs) between Sirt3 ^+/+^ and Sirt3 ^-/-^ BMDMs treated with C16:0 + MSU. (**A**) Heatmap was used to show the DEGs between Sirt3 +/+ and Sirt3 -/- BMDMs treated with C16:0 + MSU. The colors ranging from red to blue indicate the normalized levels of gene expression from high to low. (**B**) Kyoto Encyclopedia of Genes and Genomes enrichment analysis of down-regulated DEGs. (**C**) Quantification of RT-qPCR analysis of Acod1, Ccl2, Nlrc3 (Nlrp3), Itgb7, Ccl7and Csf3 mRNA expression. **Sup Fig. 4. **Viniferin treatment inhibited the expression of inflammation associated gene. After treatment of BMDMs with Viniferin and C16:0+ MSU for 12 h, cell culture supernatants and cells were collected for relevant assays. **a **Viniferin treatment reduced the Itgb7, Ccl7, Ccl2, Acod1,and Nlrc3 (Nlrp3) mRNA expression. **b** Transcription factors may bind the DNA motif of Itgb7, Ccl7,Ccl2, Acod1, and Nlrc3 (Nlrp3). **c **Viniferin treatment decreased the the protein levels of ITGB7, CCR2, NLRP3 and ACOD1. **d **Viniferin treatment inhibited CCL2 secretion. **Sup Fig. 5. **The effect of Sirt3 on Acod1 protein expression via ROS-NF-kB signaling in BMDMs treated with Mito-TEMPO (5μM) or HY-133987(1μM)and C16:0 + MSU for 12 h. **a **Mito-TEMPO treatment inhibits C16:0 + MSU crystal-induced Acod1 protein expression. **b **Mito-TEMPO treatment reversed the effect of Sirt3 deficiency on ACOD1 protein expression. **c **HY-133987 treatment prevented the impact of Sirt3 knockdown on Acod1 protein expression. **Sup Fig. 6. **These anti-inflammatory effects of Viniferin were abolished in Sirt3 deficient mice subjected to MSU-induced peritonitis. **a **The representative plots of migrated leukocytes (CD45 +) in peritoneal fluid were detected by FCM. The number of migrated leukocytes were quantified and compared among the groups on the right. **b **The levels of IL-1β and CCL2 in peritoneal fluid were detected by ELISA. n = 5 mice for each group. *P < 0.05.

## Data Availability

The data used in this study are available upon request.

## Abbreviations

MSU: Monosodium urate
PBMCs: Peripheral blood mononuclear cells
BMDMs: Bone marrow-derived macrophages
C16:0: Palmitic acid
mtROS: Mitochondrial reactive oxygen species
TCA: Tricarboxylic acid
C18:0: Stearic acid
SOD: Superoxide dismutase
AG: Acute gout
IG: Intercritical gout
ACR: American Rheumatology Association
CRP: C-reactive protein
MCE: MedChemExpress
ORF: Open reading frame
ELISA: Enzyme-linked immunosorbent assay
DEGs: Differential expression genes
qPCR: Quantitative PCR
LCM: Confocal microscope
FCM: Flow cytometer
OCR: O_2_ consumption rate
HE: Hematoxylin-eosin
SD: Standard difference
p-P65: Phosphorylation P65
KEGG: Kyoto Encyclopedia of Genes and Genomes
TEM: Transmission electron microscopy
AR: Aspect ratio
p-P65: Phosphorylation P65
