# Supplementary material for: Sirt3 improves monosodium urate crystal-induced inflammation by suppressing Acod1 expression
Source: Arthritis Res Ther. 2023 Jul 19;25:121. doi: 10.1186/s13075-023-03107-6 (PMC10354977; doi:10.1186/s13075-023-03107-6)
Supplement: Supplementary file 1 — Additional file 1: Sup Fig. 1. Sirt3 deficiency increased mitochondrial ROS production and nuclear localization of NF-kB P65in BMDMs treated with FAs + MSU crystals. a Flow analysis plotsusing FCM to detect the production of mitochondrial ROS after MitoSOX probe staining of BMDMs. b The percentages of NF-κB p65 in the BMDMsnucleus were quantified through immune-fluorescence assay. Blue shows nuclei staining with DAPI. Scale bar:40 μm. *P < 0.05. Sup Fig. 2. Sirt3 deficiency accelerated the expression of inflammation associated gene in BMDMs treated with C16:0 + MSU. a Quantitative PCR analysis was used to detect IL-1β,IL-6, TNFα, COX-2, and iNOS mRNA expression. b The protein levels of COX-2 and INOS. Sup Fig. 3. Expression analysis of differentially expressed genes (DEGs) between Sirt3 +/+ and Sirt3 -/- BMDMs treated with C16:0 + MSU. (A) Heatmap was used to show the DEGs between Sirt3 +/+ and Sirt3 -/- BMDMs treated with C16:0 + MSU. The colors ranging from red to blue indicate the normalized levels of gene expression from high to low. (B) Kyoto Encyclopedia of Genes and Genomes enrichment analysis of down-regulated DEGs. (C) Quantification of RT-qPCR analysis of Acod1, Ccl2, Nlrc3 (Nlrp3), Itgb7, Ccl7and Csf3 mRNA expression. Sup Fig. 4. Viniferin treatment inhibited the expression of inflammation associated gene. After treatment of BMDMs with Viniferin and C16:0+ MSU for 12 h, cell culture supernatants and cells were collected for relevant assays. a Viniferin treatment reduced the Itgb7, Ccl7, Ccl2, Acod1,and Nlrc3 (Nlrp3) mRNA expression. b Transcription factors may bind the DNA motif of Itgb7, Ccl7,Ccl2, Acod1, and Nlrc3 (Nlrp3). c Viniferin treatment decreased the the protein levels of ITGB7, CCR2, NLRP3 and ACOD1. d Viniferin treatment inhibited CCL2 secretion. Sup Fig. 5. The effect of Sirt3 on Acod1 protein expression via ROS-NF-kB signaling in BMDMs treated with Mito-TEMPO (5μM) or HY-133987(1μM)and C16:0 + MSU for 12 h. a Mito-TEMPO treatment inhibits C1 [file 13075_2023_3107_MOESM1_ESM.docx]

**
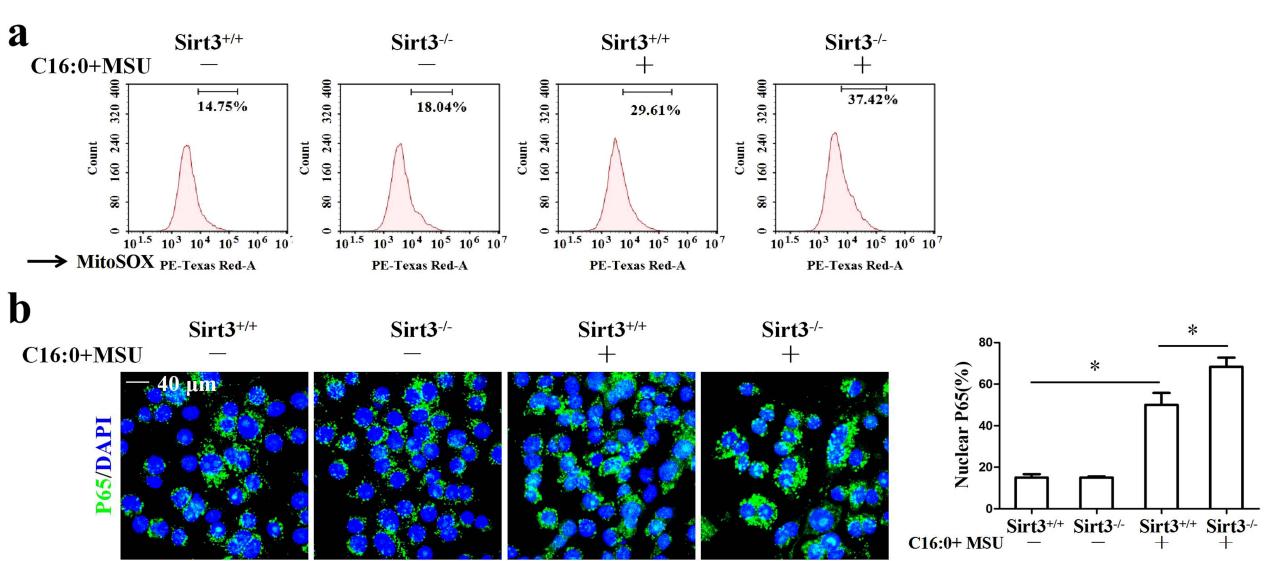
**

**Sup Fig. 1** Sirt3 deficiency increases mitochondrial ROS production and nuclear localization of NF-kB P65 in BMDMs treated with FAs + MSU crystals. **a** Flow analysis plots using FCM to detect the production of mitochondrial ROS after MitoSOX probe staining of BMDMs. **b** The percentages of NF-κB p65 in the BMDMs nucleus were quantified through immune-fluorescence assay. Blue shows nuclei staining with DAPI. Scale bar: 40 μm. **P* < 0.05.

**
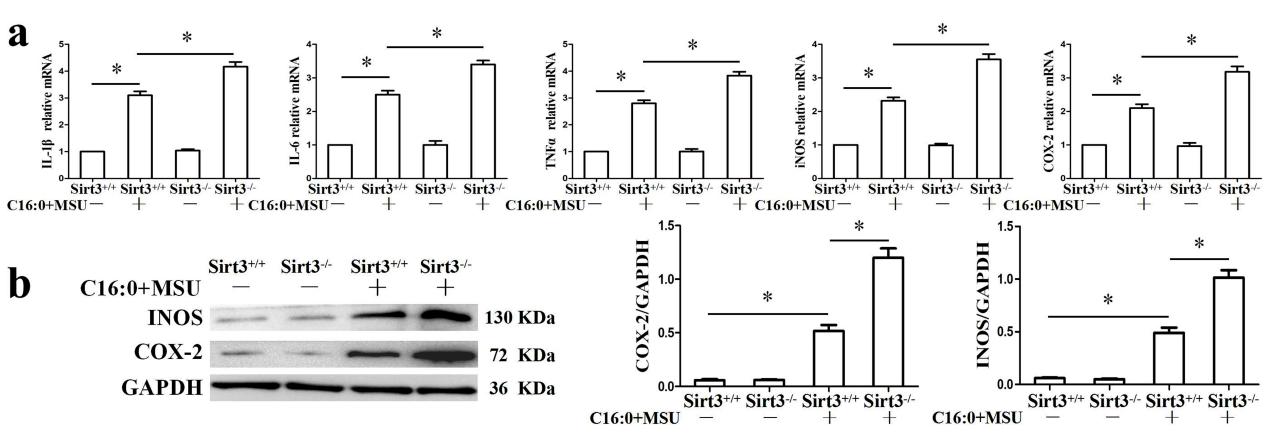
**

**Sup Fig. 2** Sirt3 deficiency accelerated the expression of inflammation associated gene in BMDMs treated with C16:0 + MSU. **a** Quantitative PCR analysis was used to detect IL-1*β*, IL-6,

TNF*α*, COX-2, and iNOS mRNA expression. **b** The protein levels of COX-2 and INOS.


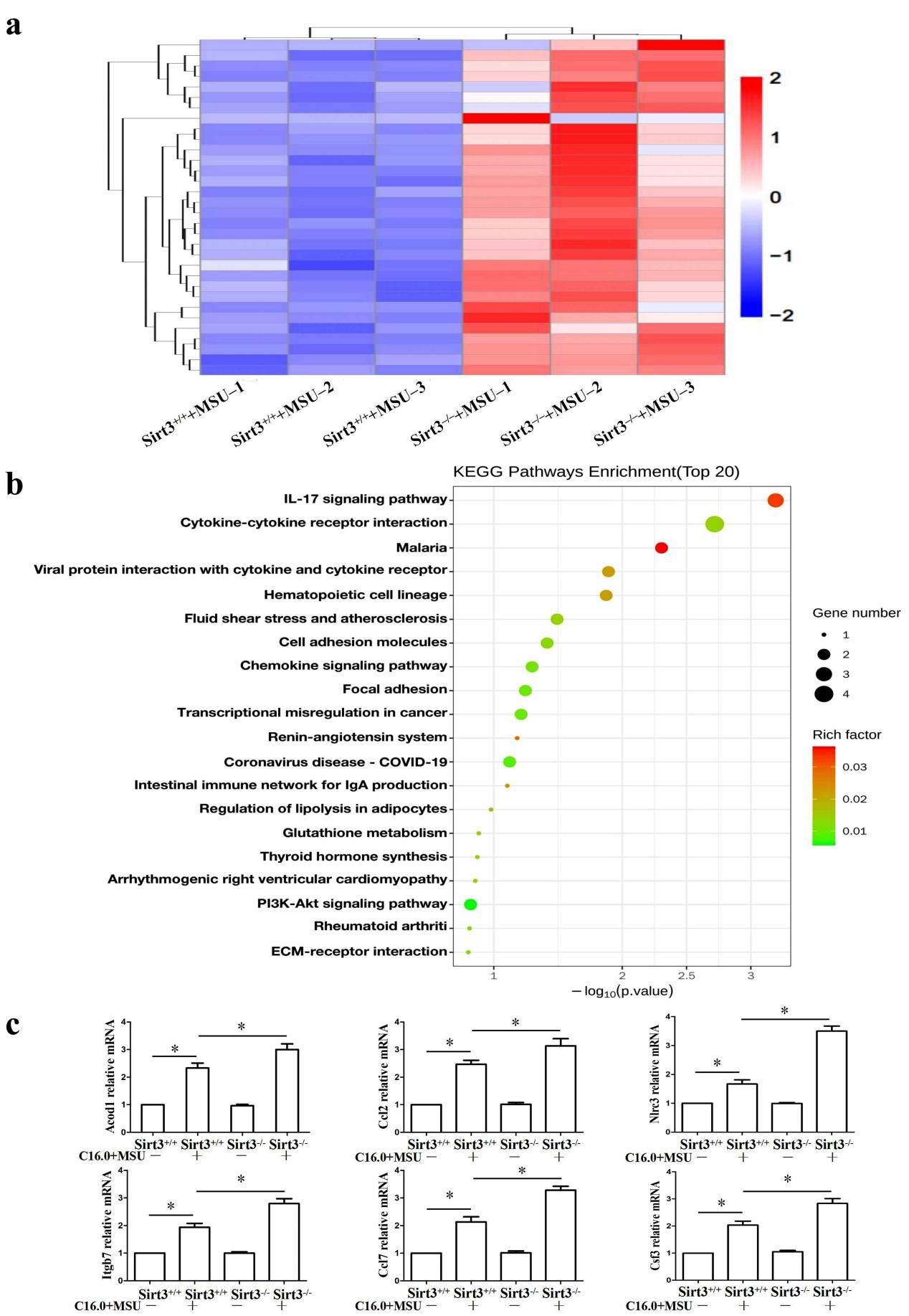


**Sup Fig. 3** Expression analysis of differentially expressed genes (DEGs) between Sirt3 +/+

and Sirt3 -/- BMDMs treated with C16:0 + MSU. (**A)** Heatmap was used to show the DEGs

between Sirt3 +/+ and Sirt3 -/- BMDMs treated with C16:0 + MSU. The colors ranging from red to blue indicate the normalized levels of gene expression from high to low. (**B)** Kyoto Encyclopedia of Genes and Genomes enrichment analysis of down-regulated DEGs. (**C)** Quantification of RT-qPCR analysis of Acod1, Ccl2, Nlrc3 (Nlrp3), Itgb7, Ccl7 and Csf3 mRNA expression.

**
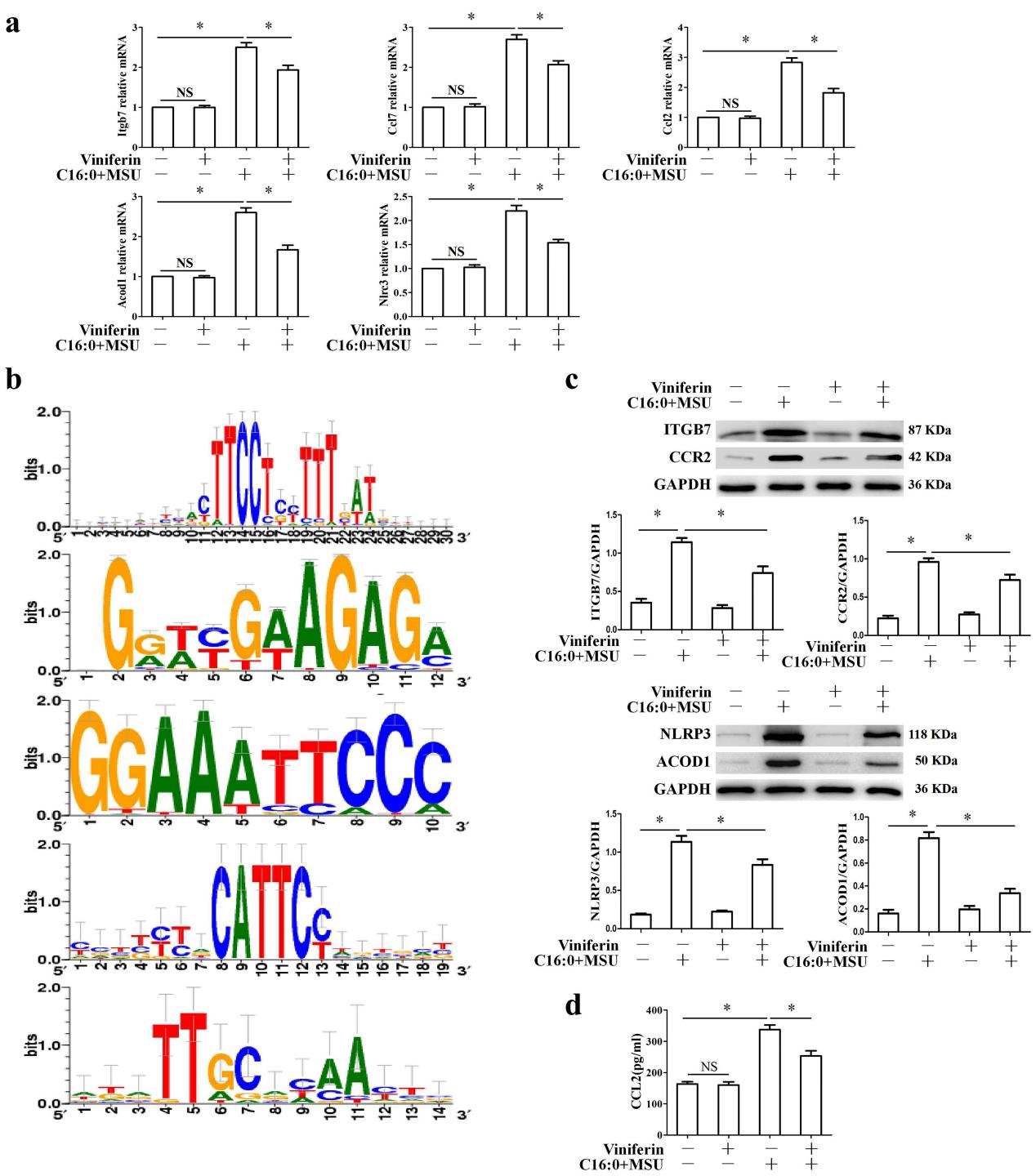
**

**Sup Fig. 4** Viniferin treatment inhibited the expression of inflammation associated gene. After

treatment of BMDMs with Viniferin and C16:0 + MSU for 12 h, cell culture supernatants and cells were collected for relevant assays. **a** Viniferin treatment reduced the Itgb7, Ccl7, Ccl2, Acod1, and Nlrc3 (Nlrp3) mRNA expression. **b** Transcription factors may bind the DNA motif of Itgb7, Ccl7, Ccl2, Acod1, and Nlrc3 (Nlrp3). **c** Viniferin treatment decreased the the protein levels of ITGB7, CCR2, NLRP3 and ACOD1. **d** Viniferin treatment inhibited CCL2 secretion.


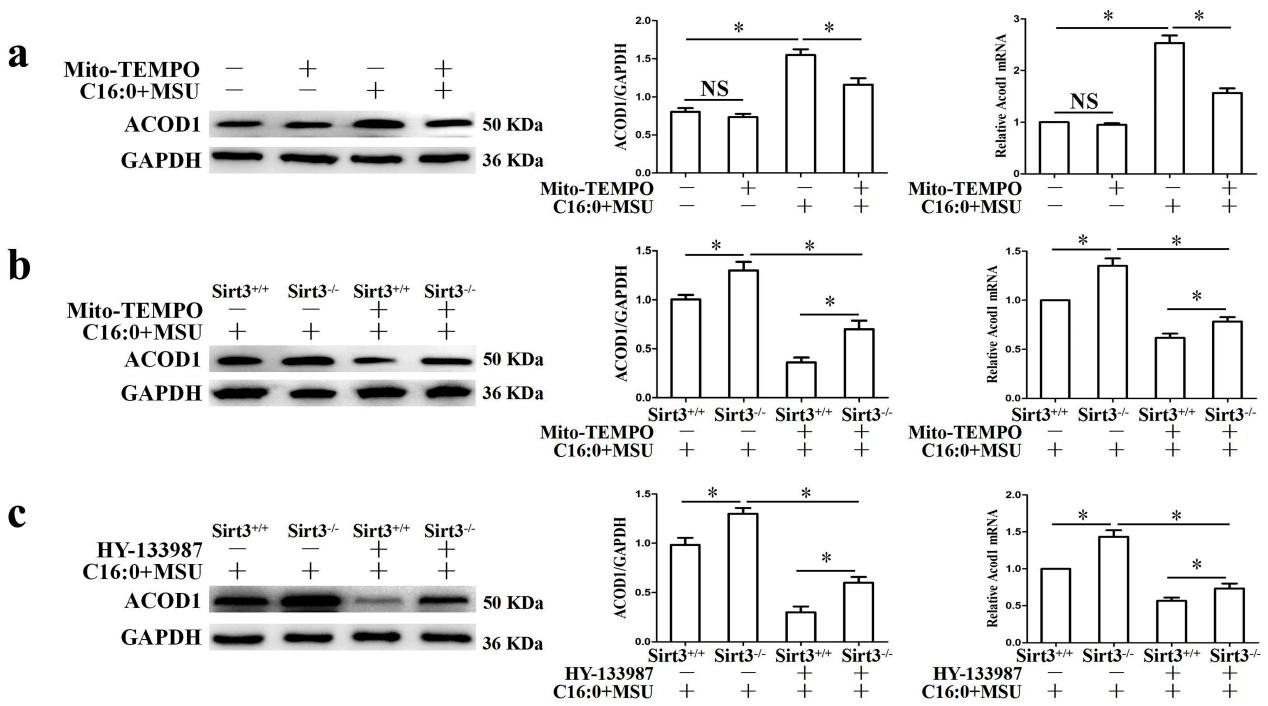


**Sup Fig. 5** The effect of Sirt3 on Acod1 protein expression via ROS-NF-kB signaling in BMDMs treated with Mito-TEMPO (5μM) or HY-133987(1μM) and C16:0 + MSU for 12 h. **a** Mito-TEMPO treatment inhibits C16:0 + MSU crystal-induced Acod1 protein expression. **b** Mito-TEMPO treatment reversed the effect of Sirt3 deficiency on ACOD1 protein expression. **c**

HY-133987 treatment prevented the impact of Sirt3 knockdown on Acod1 protein expression.


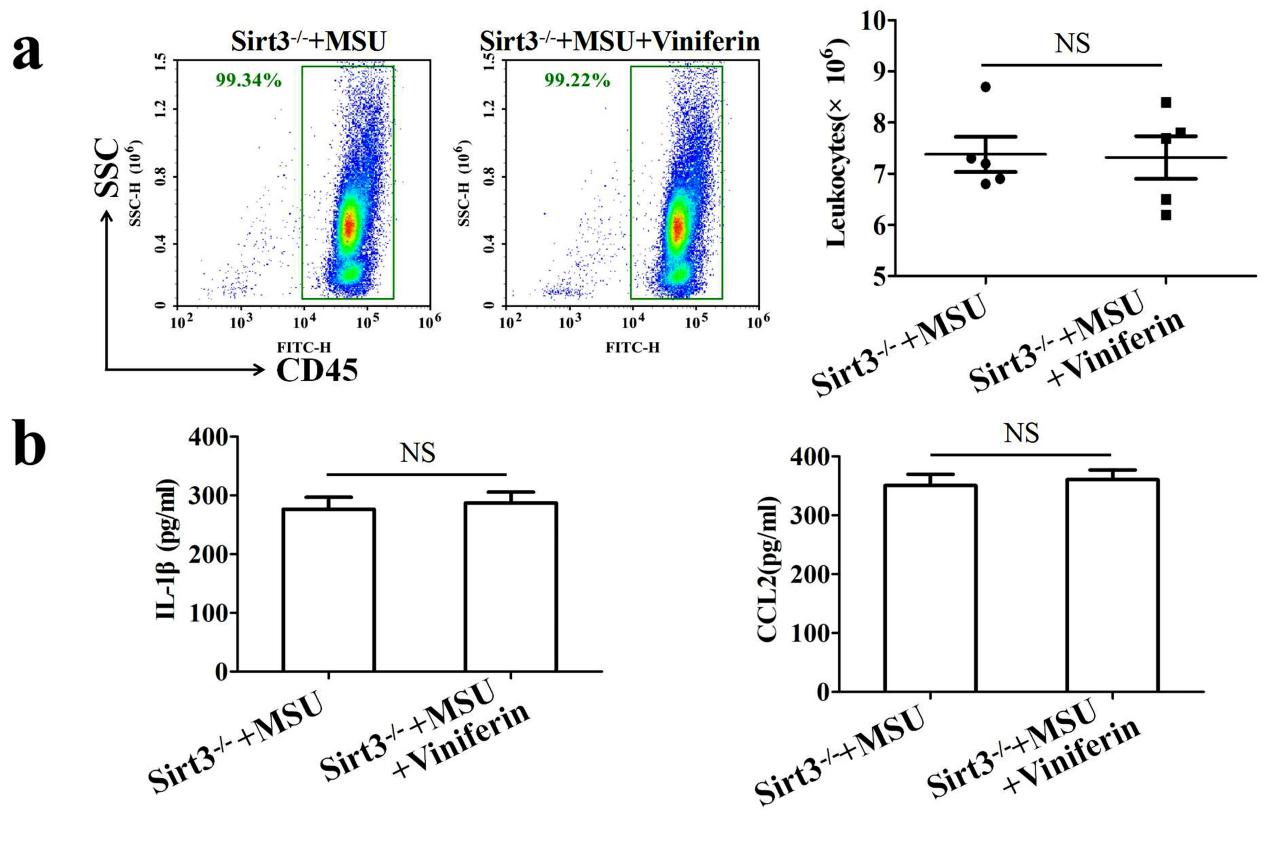


**Sup Fig. 6** These anti-inflammatory effects of Viniferin were abolished in Sirt3 deficient mice

subjected to MSU-induced peritonitis. **a** The representative plots of migrated leukocytes (CD45 + ) in peritoneal fluid were detected by FCM. The number of migrated leukocytes were quantified and compared among the groups on the right. **b** The levels of IL-1β and CCL2 in peritoneal fluid were detected by ELISA. n = 5 mice for each group. **P* < 0.05.

**The original image of Western blot**

**
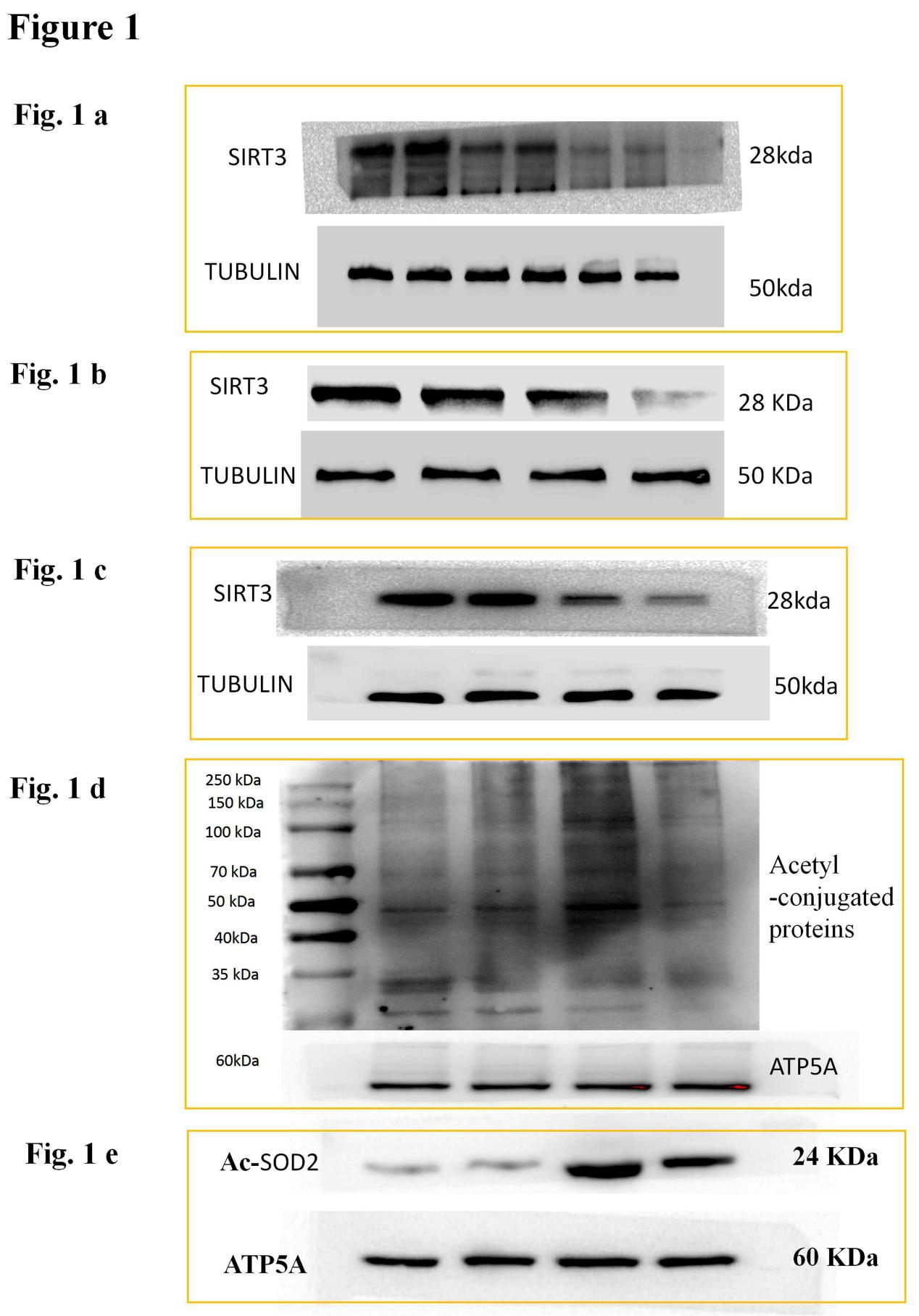
**

**
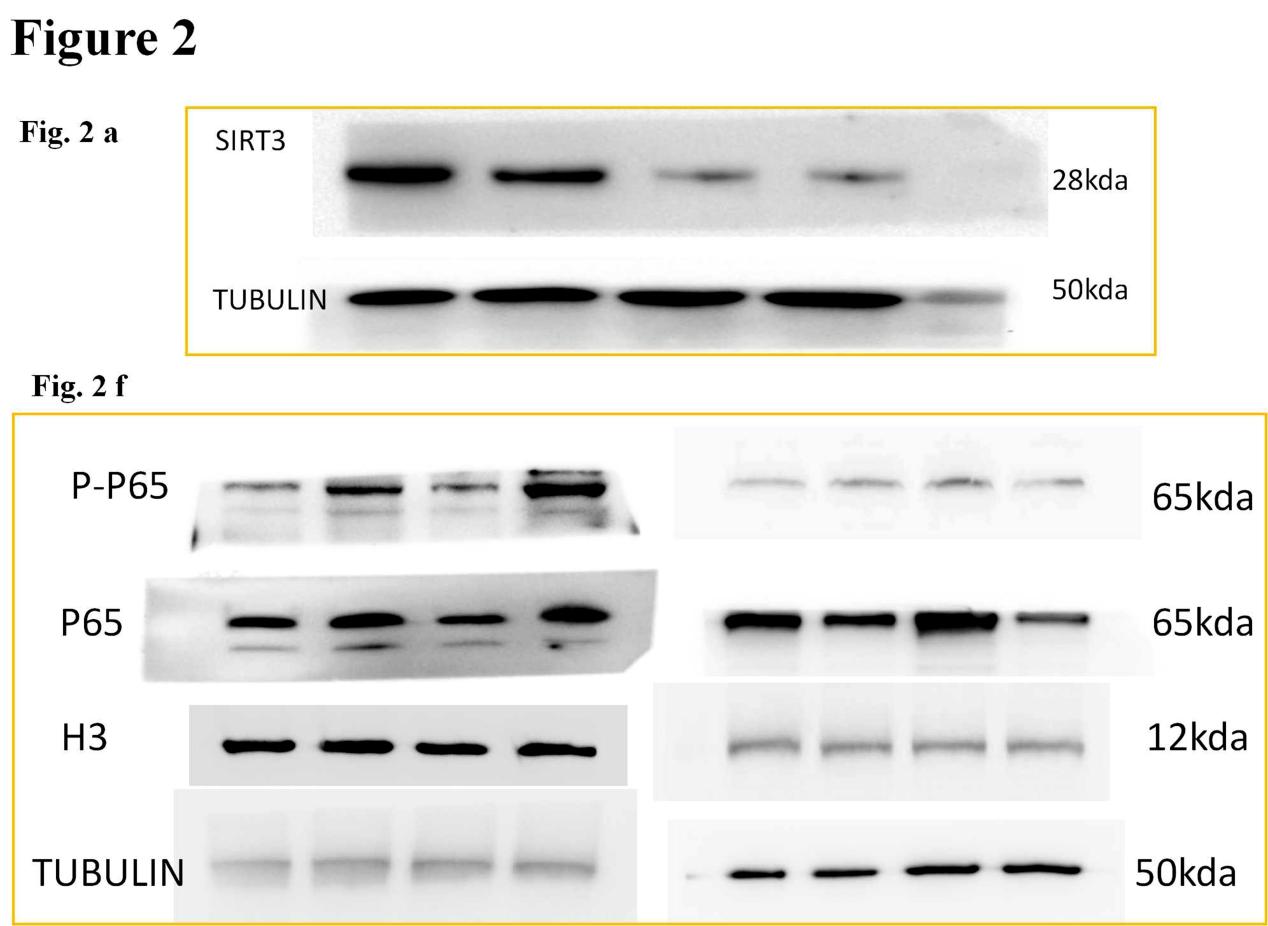
**

**
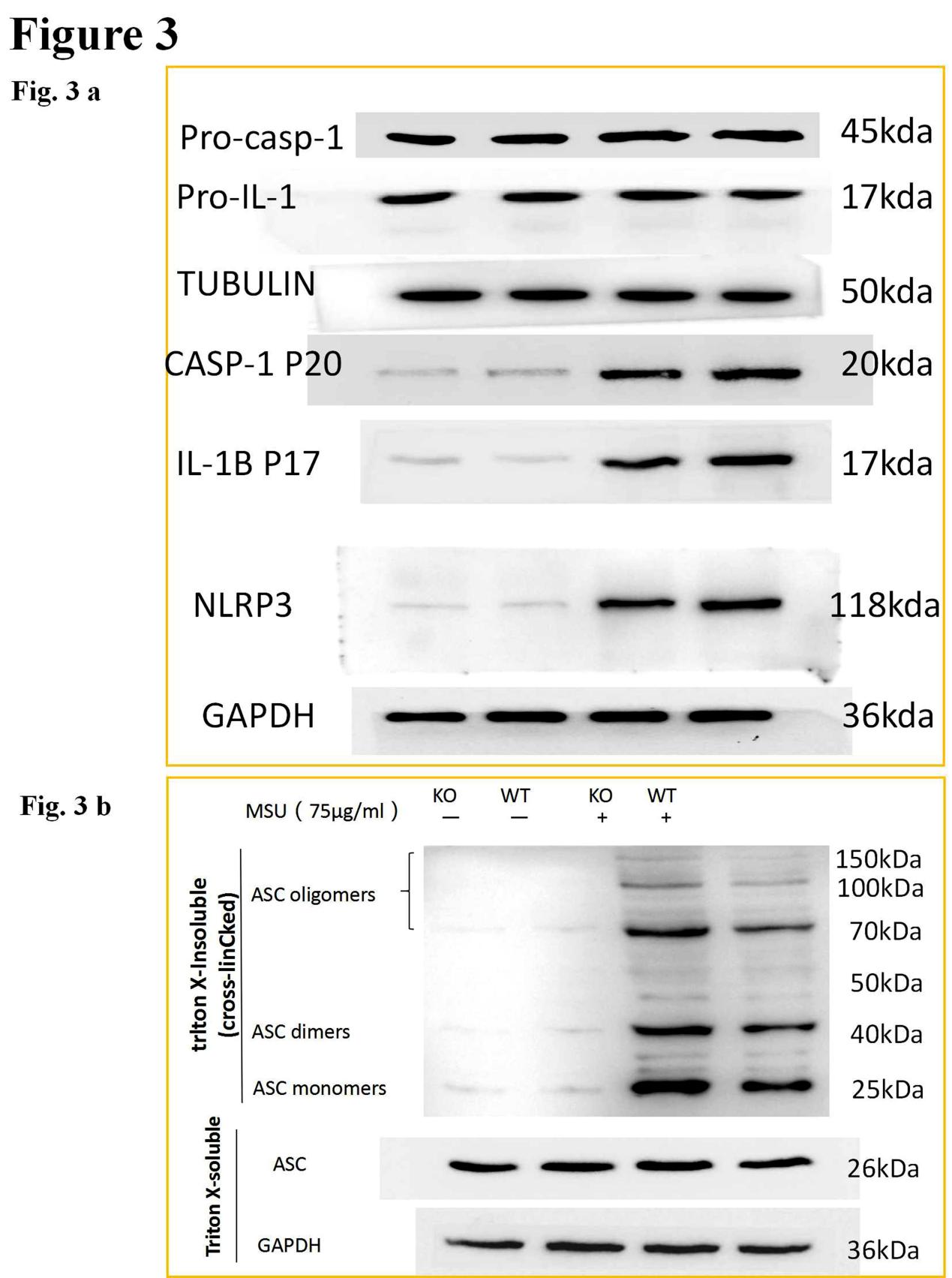
**

**
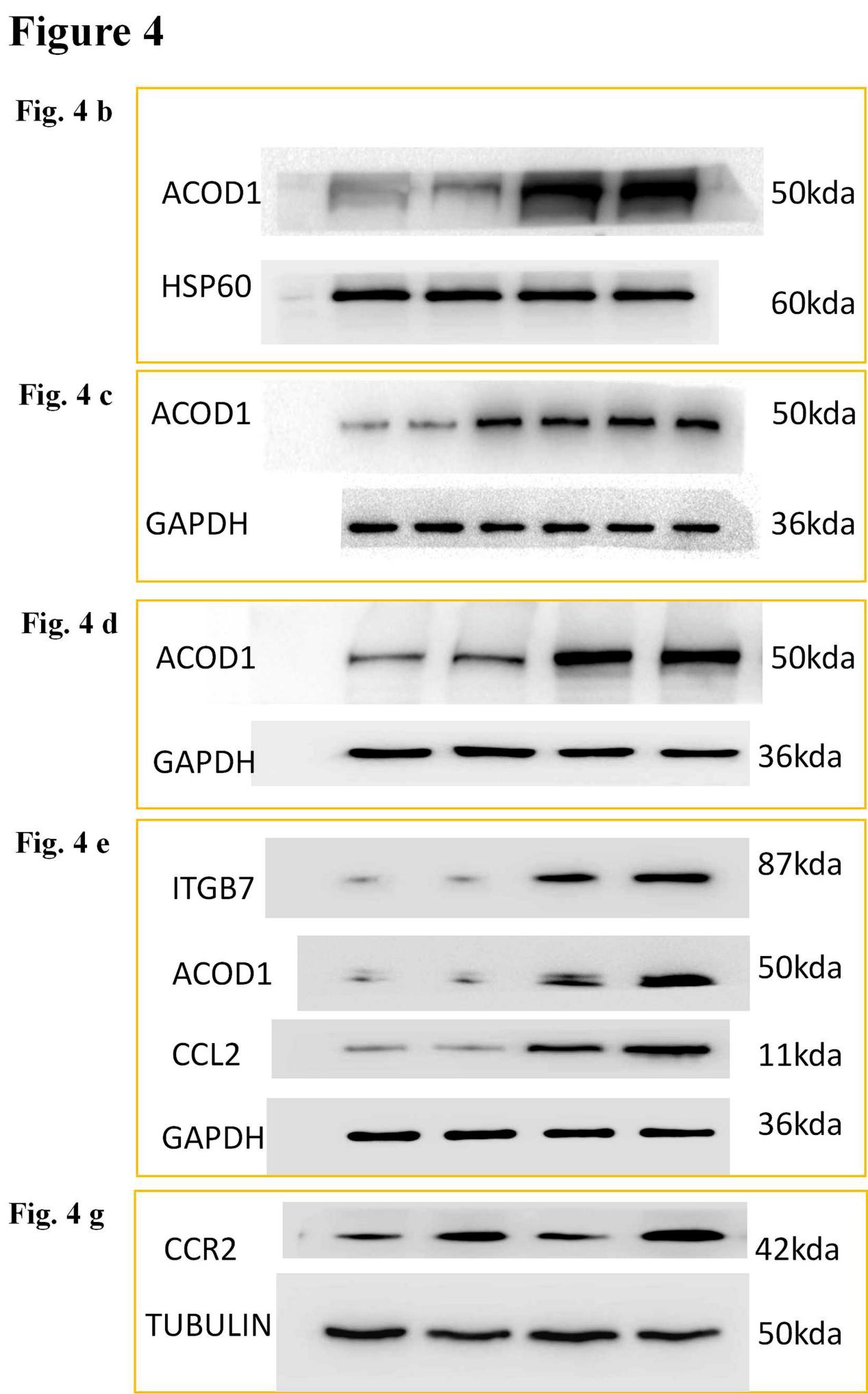
**

**
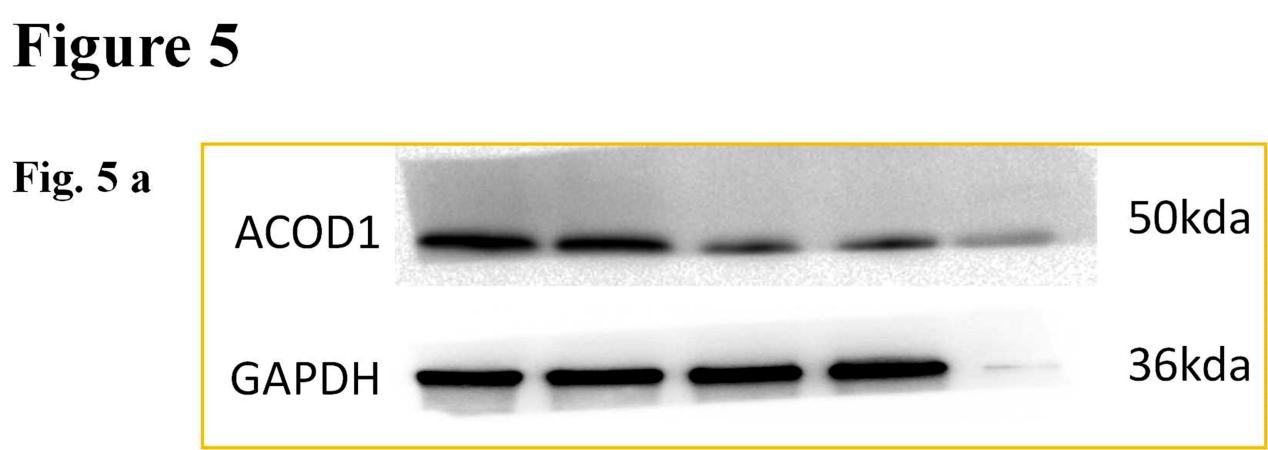
**

**
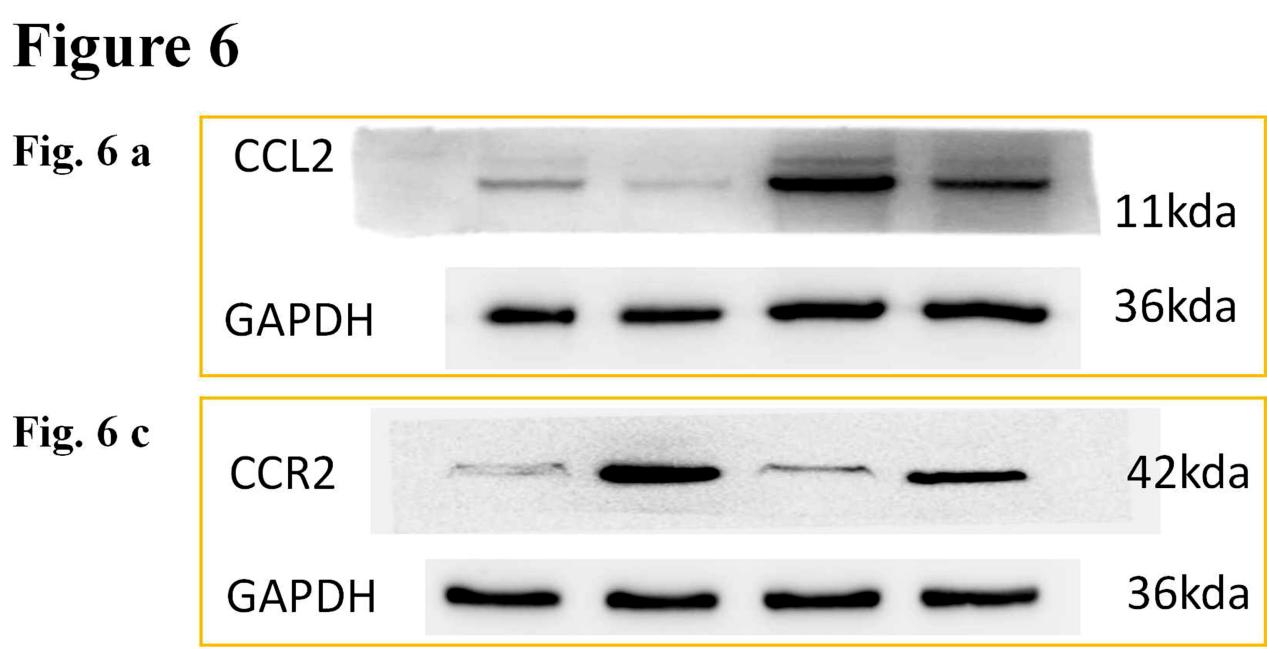
**

**
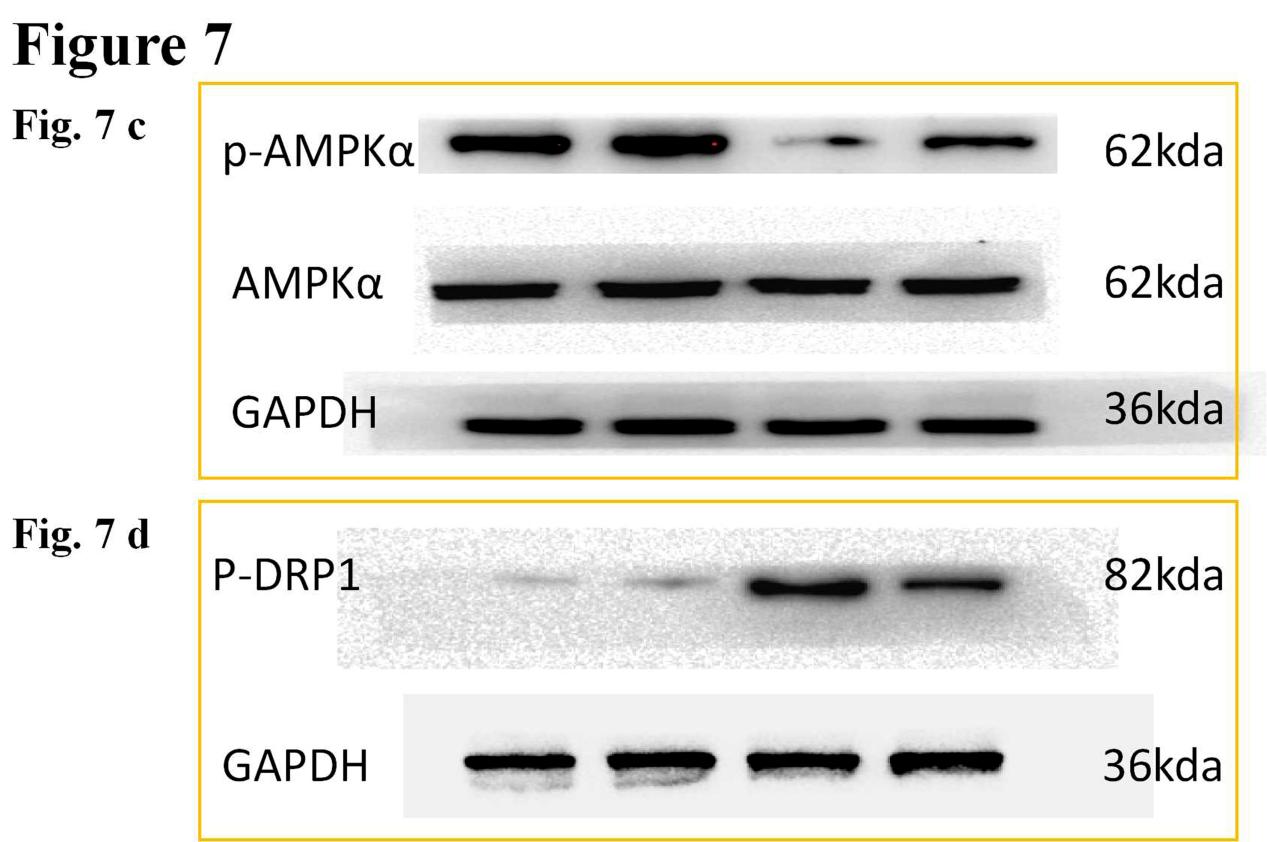
**

**
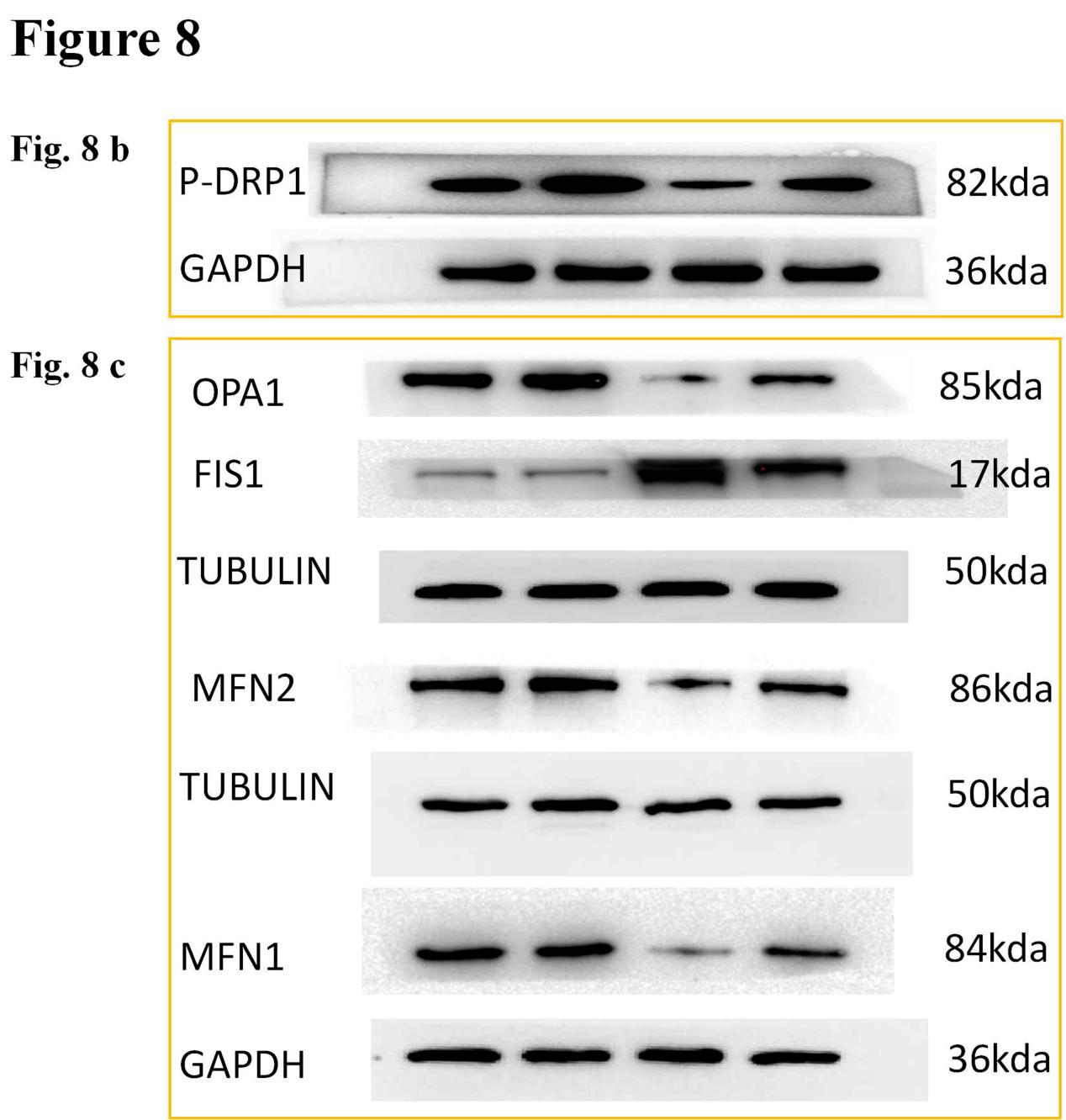
**

**
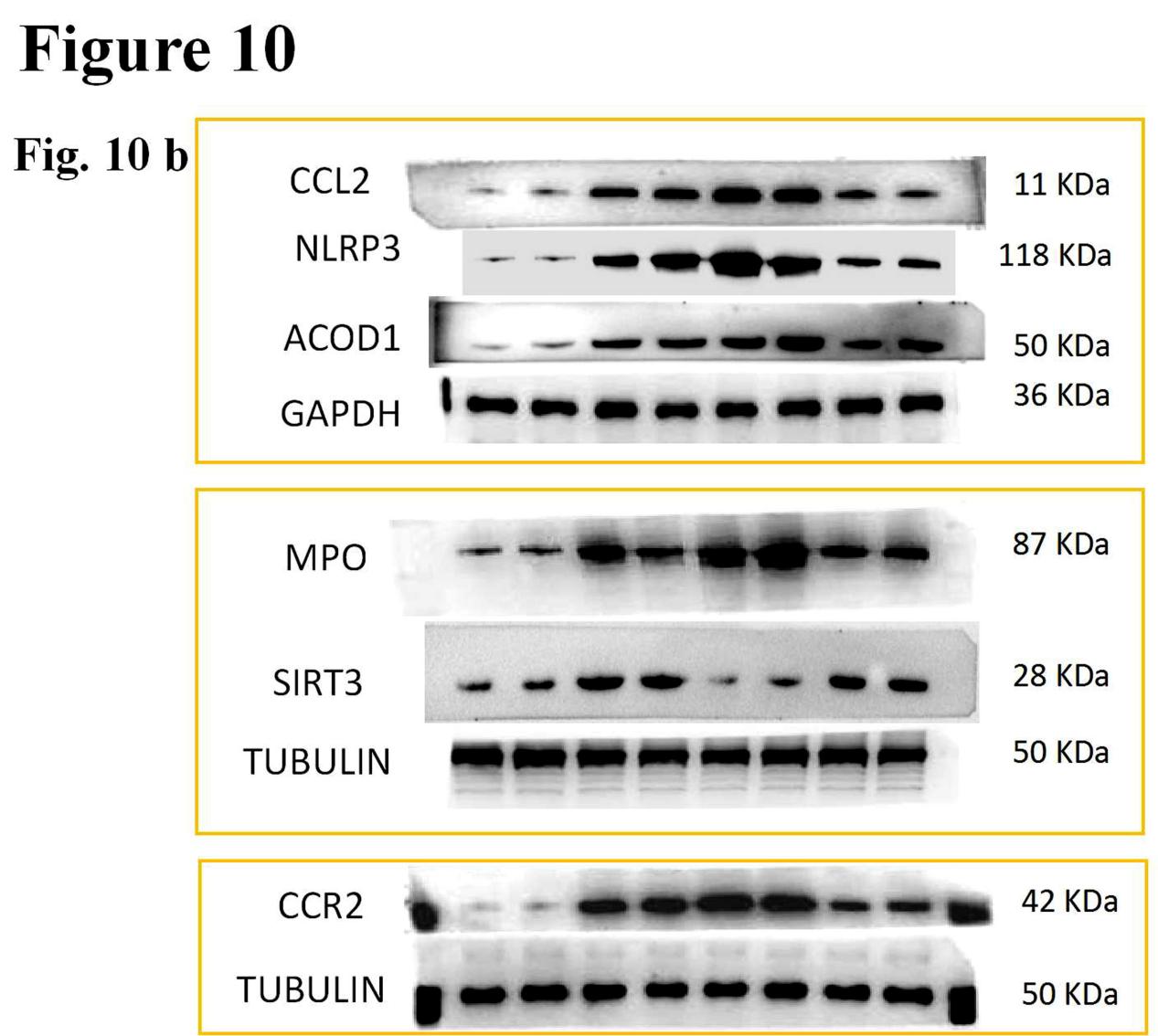
**

**
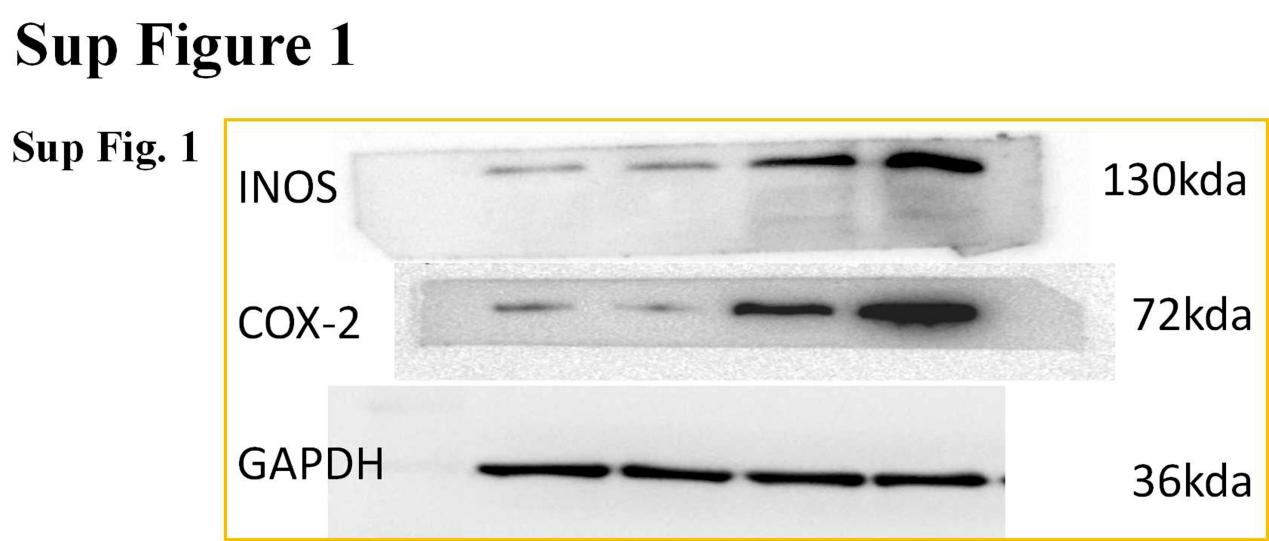
**

**
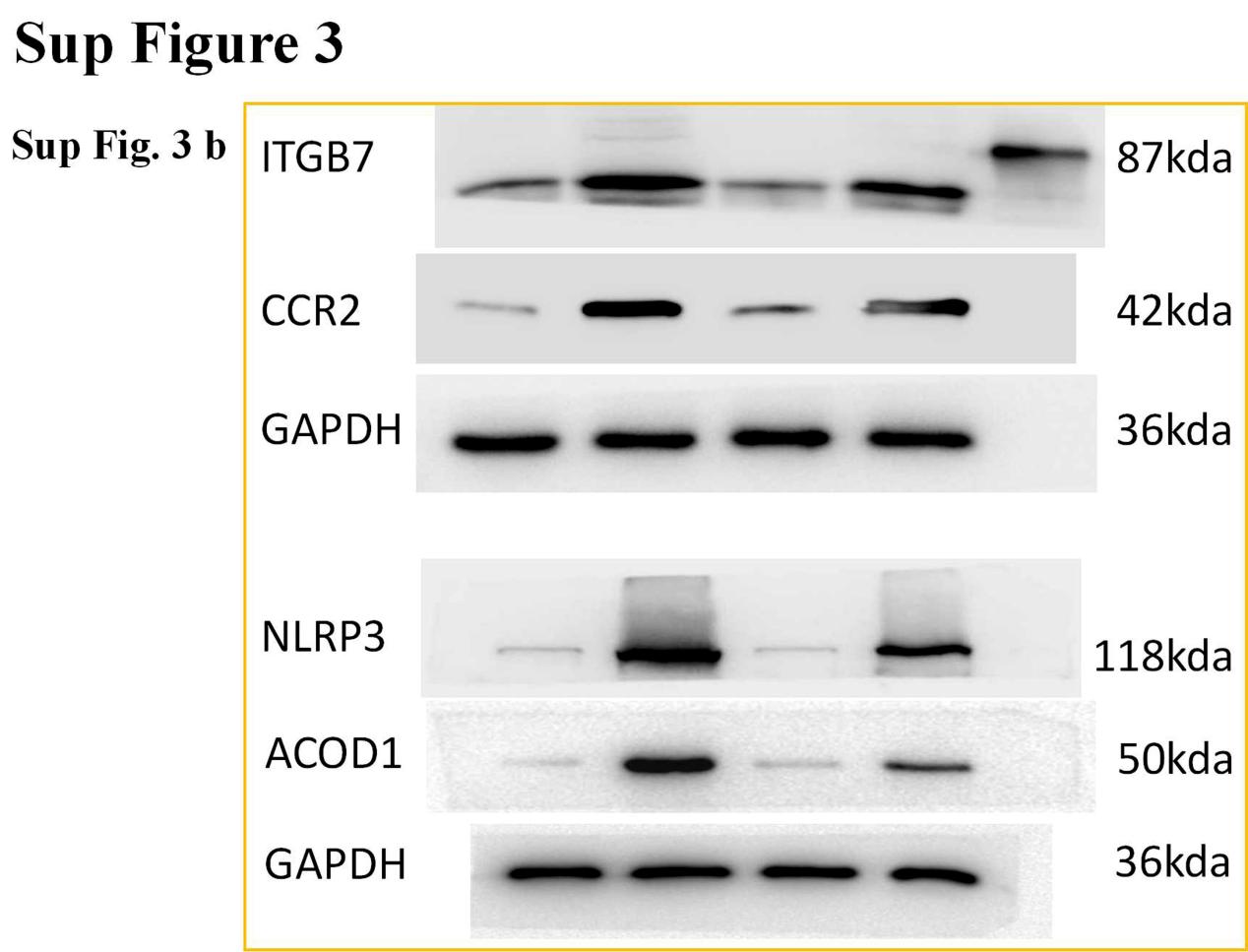
**

**
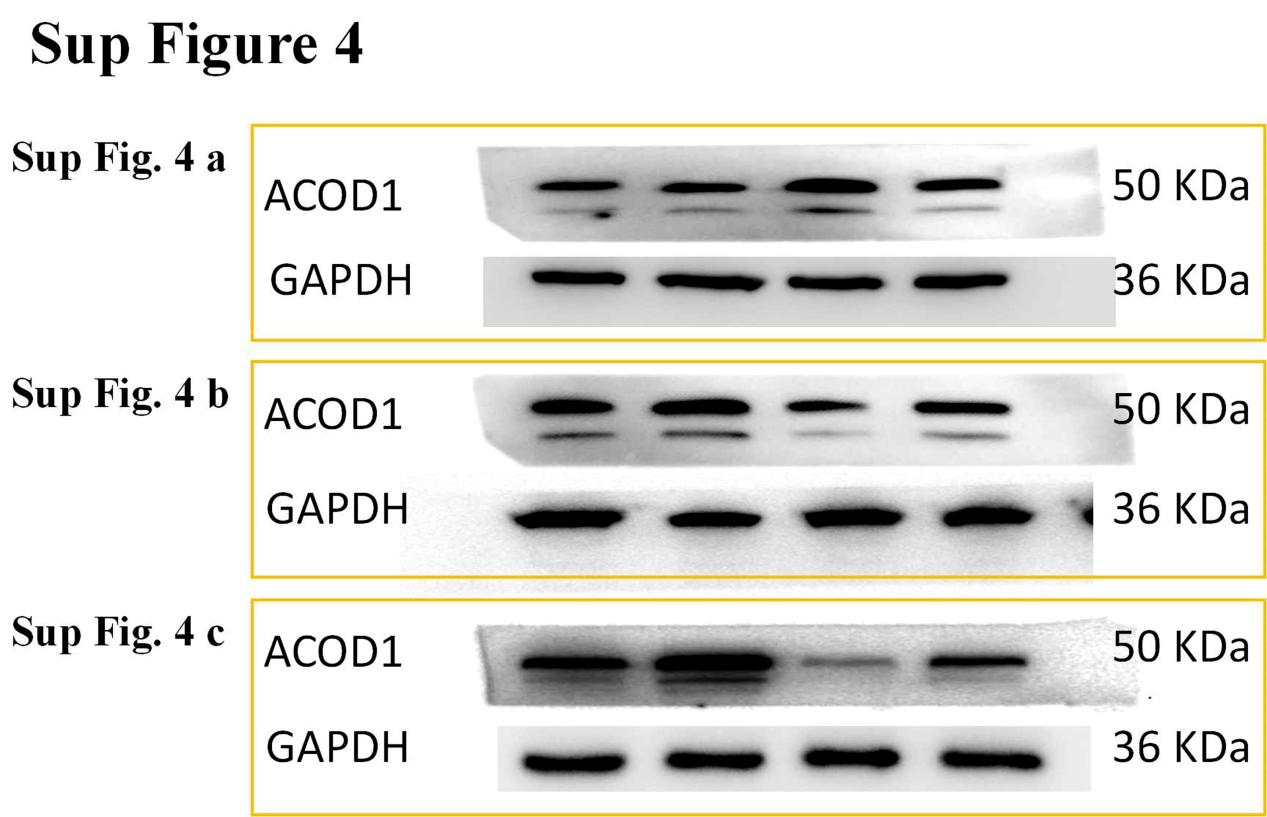
**
